# Predicting Microenvironment in CXCR4- and FAP-Positive Solid Tumors—A Pan-Cancer Machine Learning Workflow for Theranostic Target Structures

**DOI:** 10.3390/cancers15020392

**Published:** 2023-01-06

**Authors:** André Marquardt, Philipp Hartrampf, Philip Kollmannsberger, Antonio G. Solimando, Svenja Meierjohann, Hubert Kübler, Ralf Bargou, Bastian Schilling, Sebastian E. Serfling, Andreas Buck, Rudolf A. Werner, Constantin Lapa, Markus Krebs

**Affiliations:** 1 Department of Pathology, Klinikum Stuttgart, 70174 Stuttgart, Germany; 2 Department of Nuclear Medicine, University Hospital Würzburg, 97080 Würzburg, Germany; 3 Center for Computational and Theoretical Biology, University of Würzburg, 97074 Würzburg, Germany; 4 Guido Baccelli Unit of Internal Medicine, Department of Precision and Regenerative Medicine and Ionian Area-(DiMePRe-J), School of Medicine, Aldo Moro University of Bari, 70124 Bari, Italy; 5 IRCCS Istituto Tumori “Giovanni Paolo II” of Bari, 70124 Bari, Italy; 6 Institute of Pathology, University of Würzburg, 97080 Würzburg, Germany; 7 Department of Urology and Pediatric Urology, University Hospital Würzburg, 97080 Würzburg, Germany; 8 Comprehensive Cancer Center Mainfranken, University Hospital Würzburg, 97080 Würzburg, Germany; 9 Department of Dermatology, University Hospital of Würzburg, 97080 Würzburg, Germany; 10 The Russell H Morgan Department of Radiology and Radiological Science, Division of Nuclear Medicine and Molecular Imaging, Johns Hopkins School of Medicine, Baltimore, MD 21205, USA; 11 Nuclear Medicine, Medical Faculty, University of Augsburg, 86156 Augsburg, Germany

**Keywords:** machine learning, tumor microenvironment, immune infiltration, angiogenesis, mRNA, miRNA, transcriptome

## Abstract

**Simple Summary:**

Imaging based on positron emission tomography (PET) is a crucial part of up-to-date cancer care. For this purpose, PET employs and marks target structures at the cellular surface. Recently, C-X-C Motif Chemokine Receptor 4 (CXCR4) and Fibroblast Activation Protein Alpha (FAP) emerged as clinically relevant PET targets. However, it is unclear whether high levels of CXCR4 and FAP represent distinct cancer states—especially in solid tumors. Therefore, we established a machine learning model based on 9242 samples from 29 different cancer entities. Our analysis revealed that—in most solid tumors—high levels of CXCR4 were associated with immune cells infiltrating these tumors. Instead, FAP-positive tumors were characterized by high amounts of tumor vessels. Our machine learning approach potentially can identify the Achilles’ heel of tumors in a non-invasive manner—by performing PET without having to obtain tumor tissue beforehand.

**Abstract:**

(1) **Background:** C-X-C Motif Chemokine Receptor 4 (CXCR4) and Fibroblast Activation Protein Alpha (FAP) are promising theranostic targets. However, it is unclear whether CXCR4 and FAP positivity mark distinct microenvironments, especially in solid tumors. (2) **Methods:** Using Random Forest (RF) analysis, we searched for entity-independent mRNA and microRNA signatures related to CXCR4 and FAP overexpression in our pan-cancer cohort from The Cancer Genome Atlas (TCGA) database—representing *n* = 9242 specimens from 29 tumor entities. CXCR4- and FAP-positive samples were assessed via StringDB cluster analysis, EnrichR, Metascape, and Gene Set Enrichment Analysis (GSEA). Findings were validated via correlation analyses in *n* = 1541 tumor samples. TIMER2.0 analyzed the association of CXCR4 / FAP expression and infiltration levels of immune-related cells. (3) **Results:** We identified entity-independent CXCR4 and FAP gene signatures representative for the majority of solid cancers. While CXCR4 positivity marked an immune-related microenvironment, FAP overexpression highlighted an angiogenesis-associated niche. TIMER2.0 analysis confirmed characteristic infiltration levels of CD8+ cells for CXCR4-positive tumors and endothelial cells for FAP-positive tumors. (4) **Conclusions:** CXCR4- and FAP-directed PET imaging could provide a non-invasive decision aid for entity-agnostic treatment of microenvironment in solid malignancies. Moreover, this machine learning workflow can easily be transferred towards other theranostic targets.

## 1. Introduction

Positron emission tomography (PET) has become an essential part of cancer diagnostics and therapy due to its broad applicability in various cancer entities. Apart from PET-based imaging, radionuclide therapy evolved as a promising treatment option for many cancer patients—with tracers for Prostate-Specific Membrane Antigen (PSMA) and Somatostatin Receptors (SSTR) being at the forefront of this development [1,2,3]. In addition to these well-studied and clinically relevant genes, novel target structures for theranostic approaches such as C-X-C Motif Chemokine Receptor 4 (CXCR4) and Fibroblast Activation Protein Alpha (FAP) emerged, with a growing spectrum of radioligand therapies in different cancer entities [4,5]. However, there is still a lack of in-depth studies on these two genes to determine whether different expression levels actually describe distinct tumor niches or tumor microenvironments.

In malignancies, increased CXCR4 expression is associated with tumor growth, angiogenesis, and metastasis and may lead to resistance towards therapy [6]. In line with this trait, several solid cancers and hematologic malignancies exhibit CXCR4 upregulation on the cell surface [6], and radiotracer accumulation was shown to correlate with immunohistochemical CXCR4 expression of corresponding tissue samples [7]. Regarding cancer-associated fibroblasts (CAFs), membrane-bound FAP expression contributes to immune evasion and chemoresistance and appears to be crucial for invasiveness and metastasis [8,9]. Radiotracer accumulation was also shown to correlate well with immuno-histochemical FAP expression in several solid cancers [10]. Despite their potential use in a wide spectrum of cancers [7,11,12,13], it is unclear whether CXCR4 or FAP expression clearly mark distinct tumor subgroups or certain tumor microenvironments. Potentially, non-invasive CXCR4- and FAP-directed PET imaging could enable entity-agnostic diagnosis and ideally therapy, especially in solid tumors. To clarify the role of CXCR4 and FAP, we utilized a pan-cancer machine learning (ML) approach based on transcriptomic data of 29 cancer entities from The Cancer Genome Atlas (TCGA) database, searching for entity-independent mRNA and microRNA (miR) signatures best characterizing CXCR4 and FAP overexpression. In this study, we aimed to establish (for CXCR4) and evaluate (for FAP) a workflow demonstrating the utility and applicability of ML in the field of theranostics—by predicting ligand-related tumor microenvironments for other potential target structures. Of note, CXCR4- and FAP-related functions depend on a tight interaction between malignant and non-malignant cells in a certain microenvironment. Consequently, specific traits of CXCR4 and FAP cannot be exclusively attributed either to cancer cells or non-malignant immune cells or fibroblasts. However, as PET imaging also reflects the local microenvironment, we used bulk RNA data as input for our machine learning model instead of single-cell data.

Our pan-cancer ML workflow could help with characterizing target-specific tumor microenvironments and contribute to a better understanding of the basic biology of PET tracer avidity in solid tumors. These insights could also serve as a basis for further refinement of combinatorial therapeutic approaches.

## 2. Materials and Methods

### 2.1. Data Acquisition

We examined publicly available data provided by The Cancer Genome Consortium. FPKM (Fragments per Kilobase Million) files for mRNA expression and isoform quantification files for miR expression were downloaded from the GDC portal (https://portal.gdc.cancer.gov, accessed on 4 January 2022). Regarding TCGA entities, we included cohorts comprising at least 60 samples. Moreover, we did not include the READ (Rectum adenocarcinoma) cohort due to its close transcriptomic proximity to the COAD (Colon adenocarcinoma) cohort. In total, 29 of 33 available TCGA cohorts (*n* = 9242) met our inclusion criteria (Supplementary Table S1). To select specimens with a relative overexpression of CXCR4 and FAP, the respective gene was queried for each included cohort in cbioportal to eventually retrieve all samples with high expression based on the RNA-seq by expectation-maximization (RSEM, [14]) values (threshold z = 1.5) as implemented in cbioportal [15,16]. For CXCR4, 352 specimens (3.79% of the cohort) met inclusion criteria. Regarding FAP expression, 414 samples (4.47%) were included. Additionally, we assessed nine independent validation cohorts of primary tumors and metastases (*n* = 1541 samples), representing hepatocellular carcinoma, prostate cancer, renal cell carcinoma, breast cancer, and melanoma [17,18,19]. Supplementary Table S2 summarizes respective cancer entities, sample numbers, and data sources. For miRNA analysis, we used the same groups for high and low expression of CXCR4 / FAP as in the mRNA study. Protein expression data (Pan-Can 32 dataset) were downloaded from the TCPA (The Cancer Proteome Atlas) portal [20,21].

### 2.2. Machine Learning Model

Calculation was implemented in a Jupyter Notebook environment (version 7.5.0)—which is available upon request—using Python version 3.6.9, SciPy version 1.3.0 [22], and scikit-learn version 0.22.1 [23]. We applied the Random Forest (RF) Classifier (RandomForestClassifier of the sklearn.ensemble module) on unprocessed FPKM values to train 100 individual models in discriminating CXCR4- or FAP-overexpressing samples from the rest of the pan-cancer cohort, thereby adapting a procedure from a previous study [24]. Next, we split our dataset (50% training / 50% evaluation cohort), with 1000 trees in the forest (n_estimators = 1000), obtaining a mean testing accuracy of 96.37 ± 0.2% (min. 95.95%, max. 96.82%) for CXCR4 and a mean testing accuracy of 95.61 ± 0.26% (min. 95.05%, max. 96.34%) for FAP. As performed previously [24], the 200 most influential genes were determined based on the feature values of all 100 models (Supplementary Tables S3 and S4). For each model, the top 200 genes were determined, and occurrences of each gene in the top 200 were summed up. Thus, genes with the most frequent occurrences in the top 200 per model resulted in the final top 200 gene set. In analogy to the mRNA approach, we performed RF analyses for miR expression based on reads per million (RPM) miR mapped, resulting in mean testing accuracies of 96.11 ± 0.23% (min. 95.56%, max. 96.66%) and 95.51 ± 0.19% (min. 94.97%, max. 95.96%) for CXCR4 and FAP, respectively. Partial lack of miR expression data caused minor differences in sample numbers. For further in-depth analysis of the mRNA RF model, a confusion matrix was used to assess prediction results for all samples. Based on the confusion matrix, F1-measurement as well as Matthews correlation coefficients (MCC) [25] were calculated. In total, there were 8851 true negatives (TN), 195 true positives (TP), 154 false negatives (FN), and 2 false positives (FP) for CXCR4, yielding an F1 value of 0.72 and a MCC of 0.74. For FAP, the results were the following: TN = 8780, TP = 215, FN = 206, FP = 1, F1 = 0.68, MCC = 0.71.

### 2.3. Bioinformatical Analyses

We used StringDB [26] to identify potential networks between the top 200 genes. Subsequently, genes overexpressed in CXCR4/FAP-high samples (according to the mean value of expression) were analyzed using EnrichR [27,28], Metascape [29], and the “investigate gene sets” module of the Gene Set Enrichment Analysis (GSEA) webpage [30,31]. Of note, StringDB focuses on GO-Term analysis, whereas Metascape also includes Reactome and GSEA pathways. Moreover, TIMER2.0 web resource [32,33,34] was applied to determine Spearman rank correlations for CXCR4 and FAP expression with infiltration levels of immune and endothelial cells for TCGA tumor samples.

### 2.4. Literature Search Regarding MicroRNA Functions

For miRs identified within RF learning, a Google Scholar search (https://scholar.google.com, accessed on 2 March 2022) for miR-specific immune- and angiogenesis-related effects was performed. In addition, we screened four review articles [35,36,37,38] for previously reported immune-related functions (so-called ImmunomiRs) or angiogenesis-related functions (so-called AngiomiRs) of predicted miR candidates.

## 3. Results

Pan-cancer RF learning revealed a gene signature most discriminative for CXCR4 high- vs. low-expressing tumor samples (see Supplementary Table S3 for top 200 genes). Of note, CXCR4 emerged at the first position of the respective gene signature, thereby reaffirming the validity of our approach. Due to the unbalanced nature of the underlying dataset—only a minority of tumor samples strongly expressed CXCR4—we performed an internal validation step. Therefore, RF analyses were re-run without CXCR4 as gene of interest, to estimate a potential bias introduced by sample selection. The resulting top 200 gene signatures displayed an overlap with the original signatures of 90.5% (181/200 genes).

### 3.1. Signaling Pathways and Drug-Specific Signatures Related to CXCR4 Overexpression

**Figure 1:**
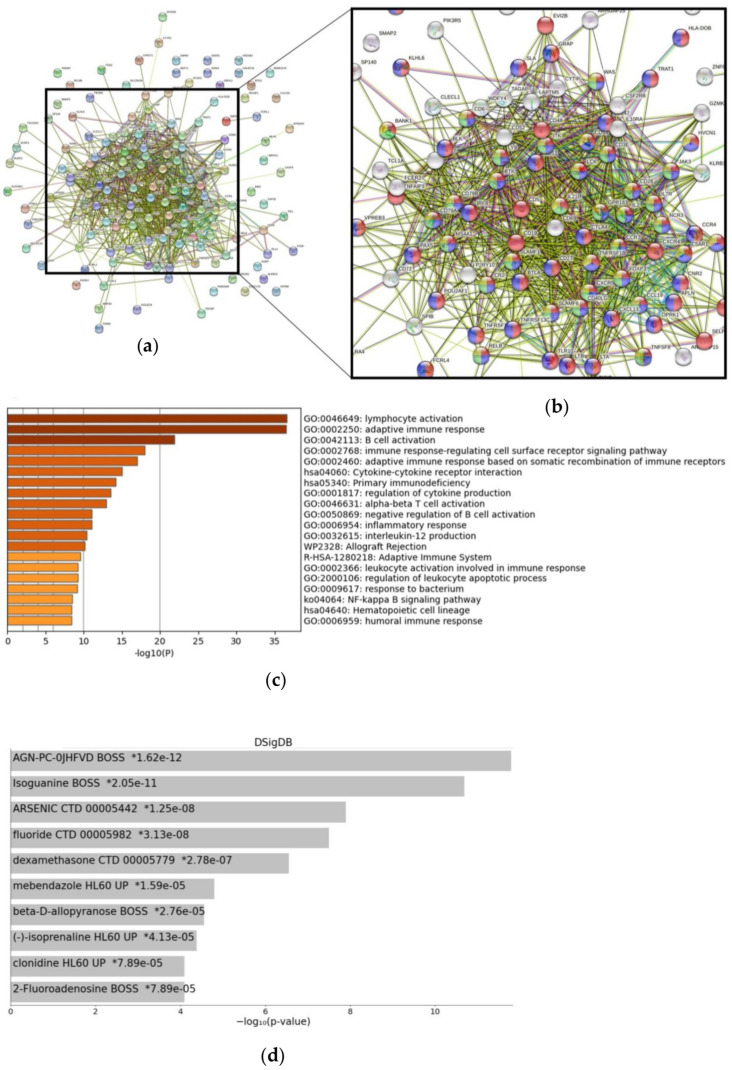
Gene networks, functions, and drug-induced gene signatures associated with CXCR4 expression. (**a**) StringDB network analysis for 141 genes included. StringDB network focused on (**b**) the main complex of the network with marked biological processes “immune system process” (GO:0002376—red), “immune response” (GO:0006955—blue), “lymphocyte activation” (GO:0046649—green), and “leukocyte activation” (GO:0045321—yellow). (**c**) Bar-graph summary of significantly enriched terms for genes overexpressed in CXCR4 high-expressing samples as provided by Metascape analysis. (**d**) Drug-induced signatures (predicted via Drug Signatures Database) significantly related to CXCR4 expression within the Random Forest learning approach. Drug Signatures Database was accessed via the EnrichR web portal.

Starting with CXCR4, StringDB analysis recognized a majority of top 200 genes as part of an immune-related cluster (Figure 1a). As illustrated in Figure 1b, genes were related to functions such as “immune system process” (GO:0002376—red), “immune response” (GO:0006955—blue), “lymphocyte activation” (GO:0046649—green), and “leukocyte activation” (GO:0045321—yellow). For Metascape analysis, we specifically selected genes from the top 200 gene list, which were overexpressed—having a significant higher mean expression according to Kruskal–Wallis test—in CXCR4 high-expressing tumor samples. In line with StringDB findings, the results confirm a highly significant role for immune-related functions (Figure 1c)—with “lymphocyte activation” (GO:0046649), “adaptive immune response” (GO:0002250), and “B cell activation” (GO:0042113) being top predicted pathways (*p* < 10^−20^). Additionally, applying the “investigate gene sets” function of the GSEA webpage to the top 200 genes further confirmed immune-related pathways as significantly overrepresented in CXCR4 high-expressing specimens (Table S5).

In a next step, we searched for drug-specific signatures (via Drug Signatures database – DSigDB [39]) characterized by a significant overlap with the CXCR4-specific gene signature. Our search revealed isoguanine, arsenic, dexamethasone, and clonidine among the top predicted therapeutic compounds (Figure 1d). While certainly requiring further in vitro validation, identified compounds could be promising candidates for future combinatorial approaches together with CXCR4-directed radioligand therapy.

### 3.2. CXCR4-Associated Tumor Microenvironment from a Pan-Cancer Perspective

**Figure 2:**
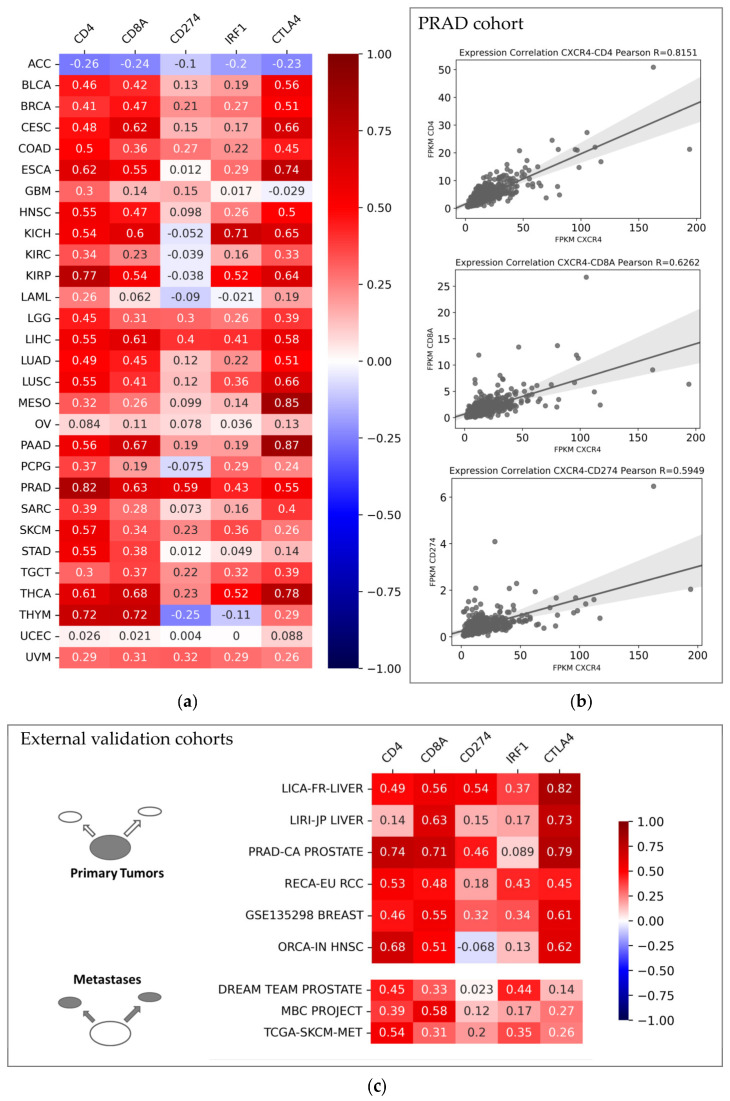
Pearson R values between CXCR4 expression and CD4, CD8A, CD274, IRF1, and CTLA4 expression within (**a**) TCGA pan-cancer cohort and (**b**) specifically within prostate cancer samples (PRAD cohort from TCGA database). (**c**) Pearson R values for respective genes in nine independent validation cohorts. FPKM: Fragments per Kilobase Million.

We further examined expression levels of CXCR4 and immune-related bona fide gene candidates within the pan-cancer cohort. In specific, we investigated the expression of the T cell co-receptors CD4 (Cluster of Differentiation 4) and CD8A (Cluster of Differentiation 8 A) as well as CD274 (Cluster of Differentiation 274, also known as PD-L1/Programed Cell Death 1 Ligand 1), IRF1 (Interferon Regulatory Factor 1), and CTLA4 (Cytotoxic T-Lymphocyte Associated Protein 4). Results of correlation analyses are presented in Figure 2a. Most cancer entities exhibited significantly positive Pearson correlations coefficients, with the highest coefficients for CD4, CD8A, and CTLA4. Entity-wise, the prostate cancer (PRAD) cohort displayed especially high correlation coefficients (Figure 2b). Beyond the TCGA database, we analyzed correlations of respective genes in nine validation cohorts from hepatocellular carcinoma; prostate, kidney, breast, and oral cancer; and melanoma. Six datasets represented primary tumors, while three datasets represented metastases. Positive Pearson R values generally confirmed TCGA results in independent datasets (Figure 2c).

**Figure 3:**
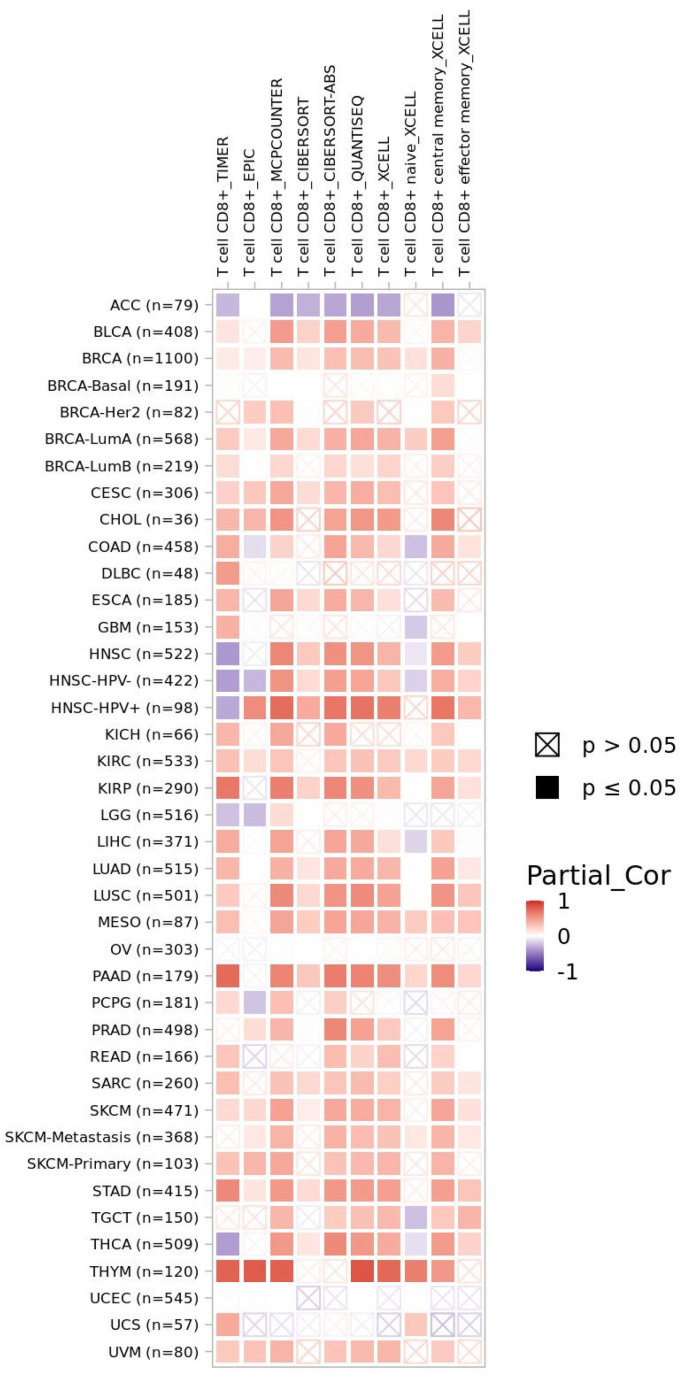
Spearman correlation coefficients of CXCR4 expression and infiltration of CD8+ T cells (and T cell subgroups) within TCGA database. Analyses were performed with TIMER2.0 web resource. Color-filled boxes represent significant results (*p* ≤ 0.05)—with red fillings indicating positive and blue fillings indicating negative correlation coefficients.

Finally, the TIMER2.0 web resource was used to investigate CD8+ T cell infiltration related to CXCR4 expression in the TCGA pan-cancer cohort (Figure 3). TIMER2.0 analysis revealed significantly positive Spearman correlation coefficients for the expression of this chemokine receptor and infiltration with CD8+ T cells (and T cell subgroups). Among cancer entities with significantly positive correlations were bladder cancer (BLCA cohort), papillary renal cell carcinoma (KIRP cohort), pancreatic adenocarcinoma (PAAD cohort), and thymoma (THYM cohort). In line with findings from transcriptomics (see Figure 2a), adrenocortical carcinoma samples from the ACC cohort displayed significantly negative correlations. Beyond CD8+ T cell infiltration, CXCR4 expression significantly correlated with B cell as well as monocyte and macrophage tissue infiltration in the majority of cancer entities investigated (Supplementary Figure S1). Of note, deviations in correlation coefficients for specific tumor entities are caused by the varying algorithms used for the estimation of immune infiltration within TIMER analyses.

### 3.3. FAP-Associated Signaling and Tumor Microenvironment

After identifying a CXCR4-associated microenvironment in solid tumors using the ML-based workflow, we aimed to validate the general applicability of the approach by analyzing the FAP-related gene signature. Of note, FAP again emerged at the top of its ML-generated gene list. As for CXCR4, we re-ran the analysis without FAP as gene of interest. The resulting top 200 gene signature yielded an overlap of 95.5% (191/200 genes) with the original gene signature.

**Figure 4:**
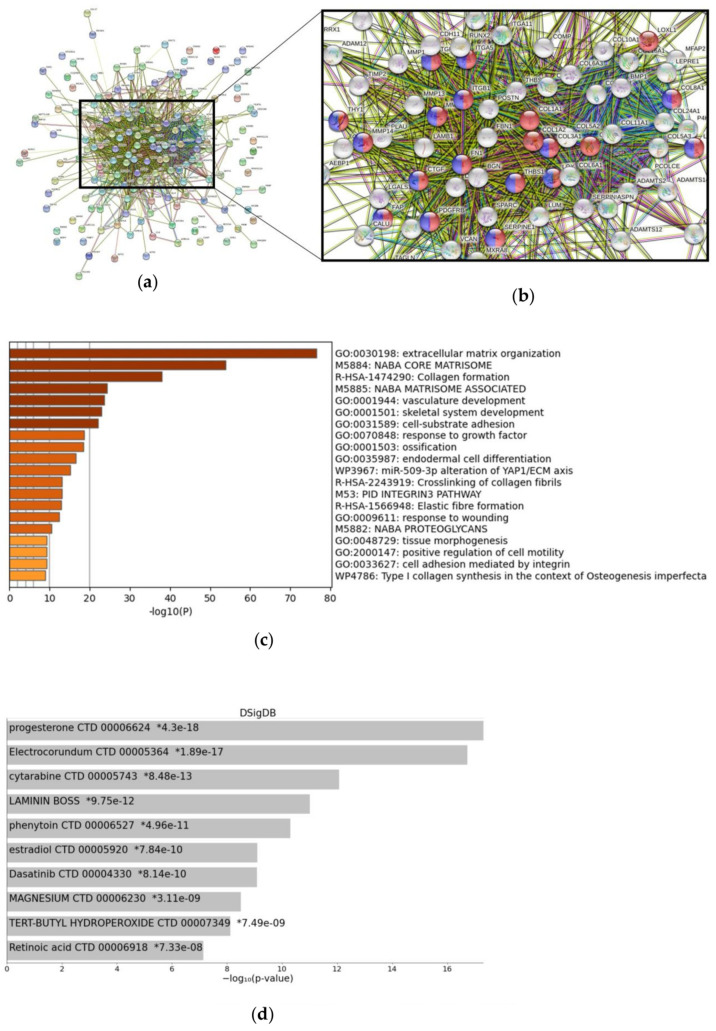
Gene networks, functions, and drug-induced gene signatures associated with FAP expression. (**a**) StringDB network analysis for 165 genes included. StringDB network focused on (**b**) the main complex of the network with marked biological processes “blood vessel development” (GO:0001568—red) and “blood vessel morphogenesis” (GO:0048514—blue). (**c**) Bar-graph summary of significantly enriched terms for genes overexpressed in FAP high-expressing samples as provided by Metascape analysis. (**d**) Drug-induced signatures (predicted via Drug Signatures Database) significantly related to FAP expression within the Random Forest learning approach. Drug Signatures Database was accessed via the EnrichR web portal.

Next, we examined FAP-related genes using the StringDB network (Figure 4a,b). Most genes were recognized as part of one cluster—representing biological processes such as “blood vessel development” (GO:0001568) and “blood vessel morphogenesis” (GO:004851). Additionally, genes from this network were involved in “extracellular matrix organization” (GO:0030198) and “collagen fibril organization” (GO:0030199) (respective genes not color-coded in Figure 4b). As shown in Figure 4c, Metascape analysis confirmed previous network analysis, with “extracellular matrix organization” as top predicted and “vasculature development” as fifth-best-predicted pathways, when considering overexpressed genes (*n* = 183) within the FAP-specific signature. We also searched for drug-specific signatures related to the top 200 FAP-related gene list. Within this approach, the agents progesterone, cytarabine, phenytoin, estradiol, and dasatinib were best predicted (Figure 4d).

**Figure 5:**
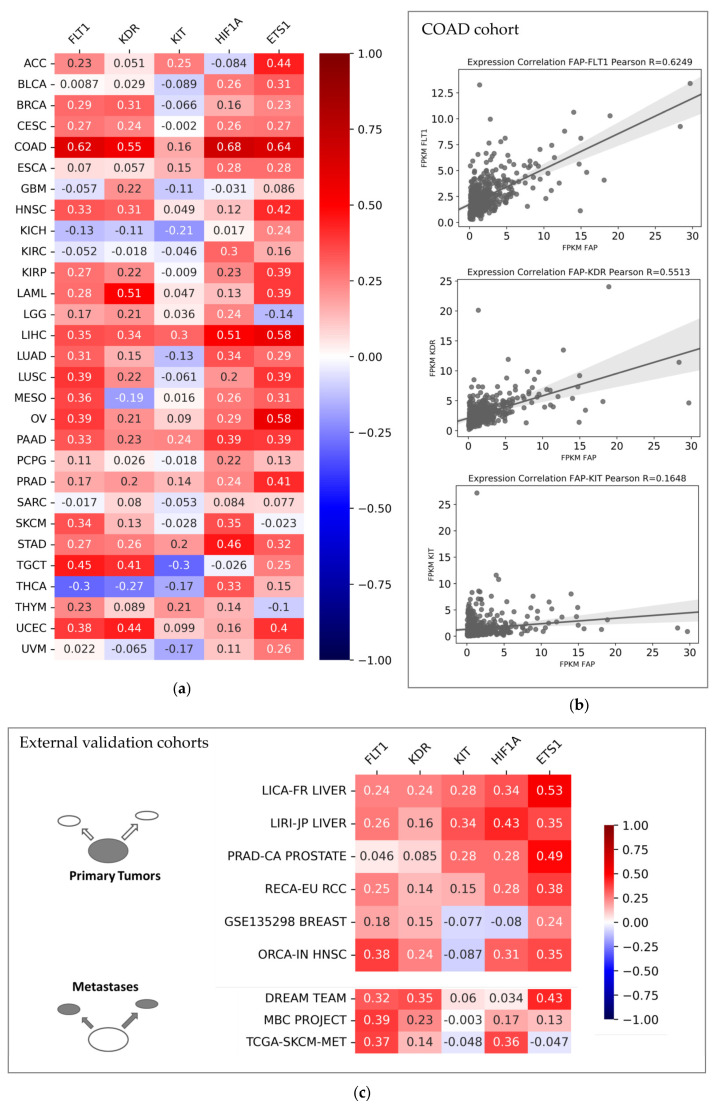
Pearson R values between FAP expression and FLT1, KDR, KIT, HIF1A, and ETS1 expression within (**a**) TCGA pan-cancer cohort and (**b**) specifically within colon adenocarcinoma (COAD cohort from TCGA database). (**c**) Pearson R values for respective genes in nine independent validation cohorts. FPKM: Fragments per Kilobase Million.

In a further step, we determined Pearson R values between FAP and selected prominent angiogenesis-related genes—specifically, FLT1 (Fms-related Receptor Tyrosine Kinase; also termed VEGFR1), KDR (Kinase Insert Domain Receptor; also termed VEGFR2), KIT (KIT Proto-Oncogene), HIF1A (Hypoxia Inducible Factor Subunit Alpha), and ETS1 (ETS Proto-Oncogene 1). As summarized in Figure 5a, we found significantly positive Pearson R values for the majority of tumor entities, especially regarding correlations between FAP and the angiogenesis-related genes FLT1, KDR, HIF1A, and ETS1. We observed the highest correlation coefficients for colon adenocarcinoma (COAD), with R = 0.62 for FAP and FLT1 and R = 0.55 for FAP and KDR. Scatter plots for the COAD cohort from TCGA are shown in Figure 5b. External validation confirmed positive correlations for FAP and angiogenesis receptors FLT1, KDR, and KIT as well as HIF1A and ETS1 in hepatocellular carcinoma but also in metastatic prostate cancer (Dream Team cohort) (Figure 5c). Of note, further in-depth analysis of correlations between FAP and angiogenesis-related genes showed mostly similar results as the previously selected bona fide candidate genes, with PDGFRB and SERPINE1 displaying the highest correlation coefficients for all entities (Figure S2).

**Figure 6:**
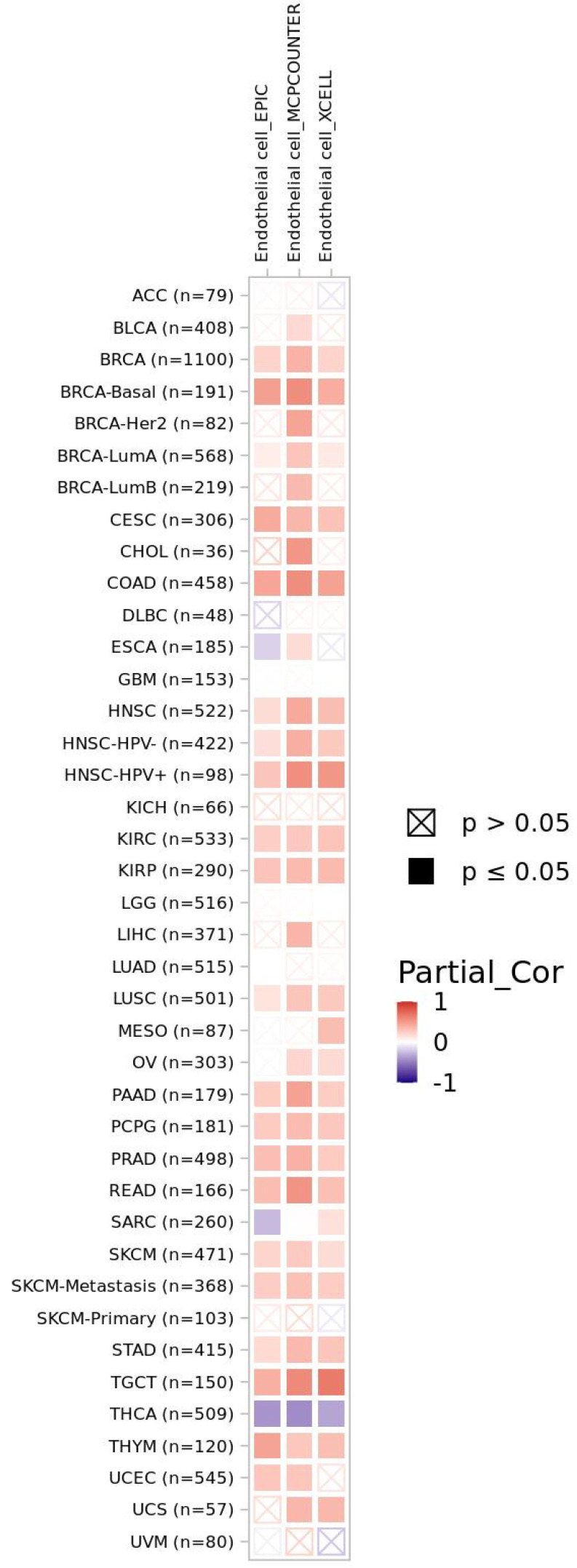
Spearman correlation coefficients of FAP expression and endothelial cell content within TCGA database. Analyses were performed with TIMER2.0 web resource. Color-filled boxes represent significant results (*p* ≤ 0.05)—with red fillings indicating positive and blue fillings indicating negative correlation coefficients.

Given the close relationship of FAP and angiogenesis-related genes, we finally looked at endothelial cell content in TCGA tumor specimens. Regarding FAP expression and endothelial cells, we also found significantly positive Spearman correlation coefficients in most tumor entities, e.g., in breast cancer (BRCA), colon adenocarcinoma (COAD), and head and neck cancer (HNSC). Analogous to transcriptomic expression analysis (see Figure 5a), thyroid carcinoma specimens (THCA) were characterized by significantly negative correlation coefficients regarding FAP expression and endothelial cell counts (Figure 6).

### 3.4. MicroRNAs Characterizing CXCR4 and FAP Overexpression

Due to the exploratory nature of our approach and based on the knowledge-confirming results of our RF models regarding the role of CXCR4 and FAP, which were characteristic for mRNA high- vs. low-expressing datasets, we wondered whether our workflow was transferable towards the miRNome. Therefore, we performed RF analyses regarding miRs best discriminating CXCR4/FAP high- vs. low-expressing tumors. The 10 best predicted CXCR4-specific miRs are summarized in Table 1. Nine out of ten top predicted miRs were previously reported to regulate the expression of immune-related target genes such as SOCS1 (Suppressor of Cytokine Signaling 1) and IRAK1 (Interleukin 1 Receptor Associated Kinase 1) [40,41,42,43,44,45,46,47,48,49,50,51,52]. As these target genes only give an impression about a small subset of effects mediated by these miRs, we additionally checked established review articles. In fact, five miRs were covered in review articles as so-called ImmunomiRs—miRs with established roles as regulators of immune pathways [35,36].

Regarding FAP-specific miRs (Table 2), eight out of ten candidates were previously reported targeting angiogenesis-related genes such as VEGFA (Vascular Endothelial Growth Factor A) and ZEB2 (Zinc Finger E-Box Binding Homeobox 2) [53,54,55,56,57,58,59,60,61,62,63,64,65,66,67,68,69]. Moreover, four miR candidates (miR-21, miR-128-2, miR-199a-1, and miR-199a-2) were previously mentioned as AngiomiRs within review articles [37,38].

### 3.5. Transferability of Transcriptomic Results to Protein Expression and Theranostics

In combination, our pan-cancer solid tumor approach showed that overexpression of CXCR4 or FAP lead to detectable transcriptional changes (in terms of mRNA and miRNA), reflected by gene signatures best distinguishing high- and low-expressing samples in RF models. Both mRNA and miR approaches confirmed previous knowledge about the impact of CXCR4 and FAP on tumor microenvironment.

To obtain an impression of how CXCR4 and FAP expression affect the protein level, we further checked bona fide candidates (Supplementary Figure S3)—CD274 (PD-L1) and CTLA4 depending on CXCR4 expression, as well as HIF1A, ETS1, and VEGFR2 depending on FAP expression. Regarding CXCR4 high-expressing samples, we observed a significant upregulation of PD-L1. Potentially due to low sample numbers available, upregulation of CTLA4 did not reach statistical significance. For HIF1A, ETS1, and VEGFR2, we detected significant protein levels in FAP-overexpressing tumor samples. However, it is important to be aware of the fact that the statistical significance is only of limited value due to the imbalance in group sizes but nevertheless indicates a certain tendency.

The analysis of a single-cell sequencing dataset representing head and neck cancer [70] clearly showed the expression variation between different cell types (Figures S4 and S5), with a significantly increased expression of CXCR4 in T cells and of FAP in fibroblasts, as expected. The bona fide candidate genes CD8A, CD4, and CD274 showed increased expression in T cells and tumor cells, respectively. Consideration of the angiogenesis-associated genes FLT1, KDR, and KIT confirms expression in endothelial cells and mast cells, respectively. However, for both datasets, basal expression of all genes also was present in tumor cells.

## 4. Discussion

Applying RF learning to transcriptomic data of 29 cancer entities, we identified the top 200 gene signatures, which were most discriminative regarding CXCR4/FAP high- vs. low-expressing tumor samples. For CXCR4, analysis recognized a majority of top 200 genes as part of an immune-related cluster. For FAP, most genes were recognized as part of biological processes such as blood vessel development and extracellular matrix organization. RF learning based on miR expression confirmed results from mRNA learning. Further analyzing transcriptomic data exhibited significantly positive Pearson correlation coefficients for most cancer entities between CXCR4 and the T cell co-receptors CD4 and CD8A, as well as IRF1 and CTLA4. For FAP, significantly positive Pearson correlations coefficients for most cancer entities were found with prominent angiogenesis-related genes FLT1 (also known as VEGFR1), KDR (also known as VEGFR2), HIF1A, and ETS1. Moreover, comparing CXCR4/FAP gene signatures with drug-induced gene signatures identified active substances such as arsenic and dexamethasone for CXCR4. Regarding FAP, progesterone and estradiol were among predicted drug candidates. After further validation, these substances could serve as potential co-therapies in combinatorial approaches targeting CXCR4- and FAP-positive tumors.

Extending our approach to the miRNome confirmed previous mRNA results, as most of the identified top 10 miRs are also well-known to regulate immune- or angiogenesis-related pathways. Taken together, concordant results from studying the transcriptome and the miRNome not only confirm previous results but also provide an (admittedly incomplete) approximation for CXCR4- and FAP-associated protein expression—when trying to transfer the results to PET avidity and theranostic applications.

### 4.1. CXCR4 as Immune-Related Biomarker in Solid Tumors

In general, enhanced CXCR4 expression seems to be associated with a worse prognosis for patients suffering from cancer. For prostate cancer, high CXCR4 levels were associated with worse cancer-related survival [71]. For colorectal as well as breast cancer, meta-analyses also confirmed poor prognosis for patients with strong CXCR4 expression [72,73].

Functionally, StringDB cluster analysis implied an entity-agnostic role for CXCR4 by identifying a common immune-related gene network. This result appears in line with previous CXCR4 research and clinical applications in hematological malignancies [11,74,75] and infections [76,77]. Of note, our cluster analysis revealed this CXCR4-specific trait based on bulk RNA expression in solid cancer tissue. In addition, nine out of ten miR candidates best describing CXCR4 overexpression within the pan-cancer cohort were reported to regulate immune-related target genes such as PD-L1 (CD274)—thereby confirming ML results based on mRNA expression. Correlation analyses confirmed results from RF learning by showing a significant co-expression of CXCR4 and immune-related genes within the TCGA database and several independent validation cohorts, especially in prostate (PRAD) and liver cancer (LIHC). Accordingly, CXCR4-overexpressing specimens from TCGA database were characterized by higher levels of infiltrating CD8+ T cells—especially in entities such as clear cell (KIRC) and papillary renal cell carcinoma (KIRP), pancreatic adenocarcinoma (PAAD), and thymoma (THYM). In summary, our pan-cancer approach showed a prominent role for CXCR4 as immune marker in solid tumors.

This role might additionally offer a new form of PET interpretation. In a broader context, CXCR4 could serve as an entity-agnostic Immuno-PET [78,79]—in order to detect an immune-related microenvironment in various malignancies. This could lead to a stratification of tumor patients for the most suitable therapy approach and avoid unnecessary therapies. In line with this potential future application, researchers and clinicians have already evaluated the effect of CXCR4 inhibition on the immune response in various tumor entities [80]. In specific, Biasci et al. investigated pancreatic and colorectal cancer and found that Plerixafor, a small molecule inhibitor of CXCR4, induced a tissue immune response [81]. In pulmonary tumors, a CXCR4-inhibiting nanocomplex led to enhanced T cell infiltration and counteracted the previous immunosuppressive microenvironment—thereby offering a rationale for a combination with an immune checkpoint blockade [82].

As an exception to the rule, adrenocortical carcinoma samples displayed negative correlations between CXCR4 expression and levels of infiltrating CD4+ and CD8+ T cells as well as PD-L1. A recent publication confirmed high tracer uptake in ACC tumors in CXCR4-directed PET/CT [83], and initial studies of immune checkpoint inhibitors in ACC were heterogeneous, with only few patients benefiting from treatment [84,85,86,87,88]. Consequently, one may speculate that CXCR4 could serve as a gatekeeper for immune checkpoint therapies in ACC. However, this assumption surely needs further investigation.

### 4.2. FAP as Potential Biomarker for Anti-Angiogenic Therapy Stratification

For FAP, intratumoral or stromal expression correlated with poor prognosis in several cancer entities, such as ovarian cancer [89], non-small cell lung cancer [90], and colorectal carcinoma [91].

StringDB analysis also detected a common gene network characteristic for FAP. Interestingly, this cluster was not only associated with fibroblast products such as collagen and extracellular matrix. Instead, angiogenesis-related signaling pathways were also associated with FAP overexpression in solid tumors. Hormones such as progesterone and estradiol were predicted to be associated with FAP overexpression within our pan-cancer cohort—with both compounds being known regulators of angiogenesis [92,93]. In the next step, we found a significant co-expression of FAP and angiogenesis-related genes for most cancer entities from our TCGA dataset and our validation datasets. The strongest evidence was found for colon adenocarcinoma (COAD) tissue. Of note, previous research described a strong relationship between FAP expression and endothelial cells in this malignancy [94]. Results from miR-based RF learning supported these results—with a majority of the top10 miR candidates reported to target crucial angiogenesis-related genes such as VEGFA and KDR. Moreover, TIMER2.0 analysis confirmed higher endothelial cell content in FAP-positive tumor samples of cancer entities such as colon adenocarcinoma (COAD) and breast cancer (BRCA). Of note, prostate cancer metastases from the Dream Team cohort also displayed relatively high correlation coefficients of FAP with FLT1 and KDR—thereby potentially mirroring the importance of angiogenesis in high-risk prostate cancer, as previously reported [95]. We hypothesize that high FAP expression in cancer patients and subsequently uptake of tracer in FAP-directed PET imaging might serve as a whole-body readout for tumor-associated angiogenesis.

### 4.3. Limitations and Future Directions

Our study surely has an exploratory character and several limitations. First, calculations are based on the TCGA database as one single data source. We aimed to reduce this bias by adding nine independent validation cohorts to our analysis. Second, transcriptomics do not automatically represent proteomics, and proteomics do not automatically represent PET tracer uptake. However, at least for FAP, a recent study implied that immunohistochemistry (IHC) results were closely associated with PET tracer uptake [10,96]. Due to the limited availability of protein expression data, we further tried to obtain a better approximation of the potential proteomic features by extending the workflow towards the miRNome, which yielded comparable results regarding CXCR4- and FAP-related microenvironment in solid tumors.

Due to the unbalanced nature of our approach (only a minority of samples represented CXCR4/FAP overexpression), we also examined F1 and MCC values. Across all tumor entities, F1 and MCC displayed a moderate overall performance of RF learning, which might be partially caused by absolute expression differences between cancer entities.

Due to the nature of the data (uneven distribution, no uniform therapies, therapy data not always available, etc.), only assumptions about the clinical relevance of CXCR4 and FAP PET-positivity can be made at this stage, which is why we intentionally refrained from looking at survival data for individual cohorts but especially in the aggregated state. This further highlights the need for studies combining PET-CT status with RNA-sequencing data. Ideally, PET images should be combined with single-cell sequencing data—to elucidate a closer look at signaling networks [97,98] shaping the tumor microenvironment.

Altogether, our approach might help open the door to a new form of PET interpretation. In a broader context, CXCR4 could be a suitable candidate for performing entity-agnostic Immuno-PET [78,79] in order to detect an immune-related microenvironment in various solid malignancies, while FAP could be a suitable candidate for detecting a microenvironment characterized by increased angiogenesis. Thus, PET-based imaging of tumor microenvironments could help with stratifying tumor patients towards most suitable therapeutic approaches while avoiding unnecessary therapies for others.

## Figures and Tables

**Table 1:** MicroRNAs (miRs) predicted to best discriminate CXCR4 high- vs. low-expressing tumor samples. *p* values significant for *p* < 0.01 are highlighted in bold.

Rank	miR Candidate	Mean High (+/−Std High)	Mean Low (+/−Std Low)	*p* Value	Immune-Related Target Genes/In Vitro (Selection)	Covered in Review Articles
1	miR-150	2204.46 (+/−3768.76)	734.17 (+/−1402.81)	1.66 × 10^−24^	c-Myb [40], ARBB2 [41]	[35,36]
2	miR-4491	1.24 (+/−2.97)	0.50 (+/−3.06)	1.87 × 10^−15^	TRIM7 [42]	-
3	miR-155	992.76 (+/−1386.74)	515.66 (+/−1180.80)	2.17 × 10^−25^	SOCS1 [43,44]	[35,36]
4	miR-5586	5.08 (+/−4.77)	3.16 (+/−3.74)	3.29 × 10^−17^	-	-
5	miR-142	7184.13 (+/−18,169.54)	4084.70 (+/−14,505.49)	1.56 × 10^−19^	PD-L1 [45]	[35,36]
6	miR-210	994.59 (+/−1418.87)	1051.44 (+/−1527.88)	0.166973729	PTPN, HOXA1, TP53I11 [46]	-
7	miR-29c	2861.48 (+/−2863.31)	2195.79 (+/−2074.10)	0.000214287	B7-H3 [47]	[36]
8	miR-195	51.30 (+/−41.85)	40.86 (+/−36.79)	4.56 × 10^−7^	PD-L1 [48,49,50]	-
9	miR-146a	544.21 (+/−2503.58)	354.89 (+/−1622.84)	3.78 × 10^−13^	IRAK1, TRAF6 [51]	[35,36]
10	miR-1307	1652.20 (+/−1937.24)	1745.57 (+/−1999.00)	0.016754954	TRAF3 [52]	-

**Table 2:** MicroRNAs (miRs) predicted to best discriminate FAP high- vs. low-expressing tumor samples. *p* values significant for *p* < 0.01 are highlighted in bold.

Rank	miR Candidate	Mean High (+/−Std High)	Mean Low (+/−Std Low)	*p* Value	Angiogenesis-Related Target Genes/In Vitro (Selection)	Covered in Review Articles
1	miR-21	305,318.37 (+/−139,932.32)	226,877.88 (+/−143,668.68)	1.81 × 10^−27^	FASLG [53], KRIT1 [54]	[37,38]
2	miR-1245a	3.78 (+/−4.81)	1.68 (+/−3.34)	2.39 × 10^−44^	-	-
3	miR-214	48.98 (+/−49.57)	28.67 (+/−63.28)	1.89 × 10^−48^	QKI [55], VEGFA [56]	-
4	miR-493	41.20 (+/−119.60)	28.61 (+/−110.12)	1.87 × 10^−42^	MIF [57,58], ZEB2 [59], DKK2 [60]	-
5	miR-128-2	73.34 (+/−93.89)	134.96 (+/−364.17)	5.27 × 10^−11^	VEGFC [61], RPS6KB1 [62]	[38]
6	miR-199a-1	1910.74 (+/−1744.58)	1190.78 (+/−2028.65)	1.26 × 10^−36^	VEGFA, VEGFR1, VEGFR2, HGF, MMP2 [63], APOE [64]	[38]
7	miR-199a-2	3107.70 (+/−2798.14)	1940.26 (+/−3134.63)	2.47 × 10^−35^	VEGFA [65], APOE [64]	[38]
8	miR-652	30.18 (+/−36.47)	36.92 (+/−41.11)	7.14 × 10^−10^	VEGFA [66], PRRX1 [67]	-
9	miR-337	61.72 (+/−130.58)	51.09 (+/−152.74)	1.69 × 10^−37^	-	-
10	miR-7-1	25.56 (+/−31.47)	40.47 (+/−65.27)	6.98 × 10^−10^	KLF4 [68], RAF1 [69]	-

## Data Availability

All datasets in this study are publicly available and were accessed either via GDC-portal (https://portal.gdc.cancer.gov/projects, accessed on 4 January 2022) or cBioPortal (https://www.cbioportal.org/, accessed on 4 January 2022). The Jupyter Notebook containing all calculations is available upon request from A.M. (a.marquardt@klinikum-stuttgart.de).

## Supplementary Materials

The following supporting information can be downloaded at: https://www.mdpi.com/article/10.3390/cancers15020392/s1, Figure S1: Spearman correlation coefficients of CXCR4 expression and infiltration of B cells (**a**), monocytes (**b**), and macrophages (**c**) within TCGA database. Figure S2: Protein expression depending on CXCR4/FAP high vs. low expression in the TCGA database. Table S1: Proportion of tumor specimens overexpressing CXCR4 or FAP—according to underlying tumor entities from the TCGA (The Cancer Genome Atlas) database. Figure S3: Protein expression depending on CXCR4/FAP high vs. low expression in the TCGA database. Figure S4: Cell-type specific expression of CXCR4 and a selection of immune-related genes within a single cell sequencing dataset representing head and neck cancer (GSE103322). Figure S5: Cell-type specific expression of FAP and a selection of angiogenesis-related genes within a single cell sequencing dataset representing head and neck cancer (GSE103322). Table S1: Proportion of tumor specimens overexpressing CXCR4 or FAP – according to underlying tumor entities from the TCGA (The Cancer Genome Atlas) database. Table S2: Independent validation cohorts with respective cancer entities, sample numbers, and data sources. Table S3: Top 200 genes identified by RF learning being most discriminative for CXCR4 high- vs. CXCR4 low-expressing cancer samples. Table S4: Top 200 genes identified by RF learning being most discriminative for FAP high- vs. FAP low-expressing cancer samples. Table S5: CXCR4- (A) and FAP-specific (B) gene signatures analyzed using the “investigate gene sets” module of the Gene Set Enrichment Analysis (GSEA) webpage. FDR: false discovery rate.
