# Supplementary material for: Predicting Microenvironment in CXCR4- and FAP-Positive Solid Tumors—A Pan-Cancer Machine Learning Workflow for Theranostic Target Structures
Source: Cancers (Basel). 2023 Jan 6;15(2):392. doi: 10.3390/cancers15020392 (PMC9856808; doi:10.3390/cancers15020392)

Supplementary Material

Table S1. Proportion of tumor specimens overexpressing CXCR4 or FAP – according to underlying tumor entities from the TCGA (The Cancer Genome Atlas) database.

TCGA ID	Tumor Entity	CXCR4	FAP
**ACC**	Adrenocortical carcinoma	6/79 (7.59%)	3/79 (3.79%)
**BLCA**	Bladder Carcinoma	9/408 (2.20%)	17/408 (4.16%)
**BRCA**	Breast Carcinoma	36/1090 (3.30%)	37/1090 (3.39%)
**CESC**	Cervical Carcinoma	10/304 (3.28%)	18/304 (5.92%)
**COAD**	Colon Adenocarcinoma	13/285 (4.56%)	16/285 (5.61%)
**ESCA**	Esophageal Carcinoma	2/164 (1.21%)	12/164 (7.31%)
**GBM**	Glioblastoma Multiforme	6/159 (3.77%)	7/159 (4.40%)
**HNSC**	Head and Neck Squamous Cell Carcinoma	19/500 (3.8%)	22/500 (4.39%)
**KICH**	Kidney Chromophobe	2/66 (3.03%)	1/66 (1.51%)
**KIRC**	Kidney Renal Clear Cell Carcinoma	26/530 (4.90%)	30/530 (5.66%)
**KIRP**	Kidney Renal Papillary Cell Carcinoma	12/289 (4.15%)	14/289 (4.84%)
**LAML**	Acute Myeloid Leukemia	9/145 (6.20%)	4/145 (2.75%)
**LGG**	Brain Lower Grade Glioma	27/511 (5.28%)	20/511 (3.91%)
**LIHC**	Liver Hepatocellular Carcinoma	18/371 (4.85%)	16/371 (4.31%)
**LUAD**	Lung Adenocarcinoma	13/513 (2.53%)	15/513 (2.92%)
**LUSC**	Lung Squamous Cell Carcinoma	16/501 (3.19%)	24/501 (4.79%)
**MESO**	Mesothelioma	2/86 (2.32%)	7/86 (8.13%)
**OV**	Ovarian Serous Cystadenocarcinoma	16/265 (6.03%)	21/265 (7.92%)
**PAAD**	Pancreatic Adenocarcinoma	5/177 (2.82%)	10/177 (5.64%)
**PCPG**	Pheochromocytoma and Paraganglioma	8/179 (4.46%)	2/179 (1.11%)
**PRAD**	Prostate Adenocarcinoma	17/495 (3.43%)	23/495 (4.64%)
**SARC**	Sarcoma	11/259 (4.24%)	11/259 (4.24%)
**SKCM**	Skin Cutaneous Melanoma	9/468 (1.92%)	21/468 (4.48%)
**STAD**	Stomach Adenocarcinoma	14/376 (3.72%)	15/376 (3.98%)
**TGCT**	Testicular Germ Cell Tumors	9/150 (6.0%)	3/150 (2.0%)
**THCA**	Thyroid Carcinoma	20/498 (4.01%)	24/498 (4.81%)
**THYM**	Thymoma	4/119 (3.36%)	5/119 (4.20%)
**UCEC**	Uterine Corpus Endometrial Carcinoma	8/175 (4.57%)	13/175 (7.42%)
**UVM**	Uveal Melanoma	4/80 (5.0%)	3/80 (3.75%)
		**351/9,242 (3.79%)**	**414/9,242 (4.47%)**

Table S2. Independent validation cohorts with respective cancer entities, sample numbers and data sources.

Name	Tumor Entity	n	data source
**LICA-FR**	Liver / Hepatocellular Carcinoma (HCC)	161	https://dcc.icgc.org/projects/LICA-FR
**LIRI-JP**	Liver / Hepatocellular Carcinoma (HCC)	232	https://dcc.icgc.org/projects/LIRI-JP
**PRAD-CA**	Prostate Adenocarcinoma	144	https://dcc.icgc.org/projects/PRAD-CA
**RECA-EU**	Renal Cell Carcinoma (RCC)	91	https://dcc.icgc.org/projects/RECA-EU
**GSE135298**	Breast Cancer	93	(16)
**ORCA-IN**	Head and Neck Squamous Cell Carcinoma (HNSC)	40	https://dcc.icgc.org/projects/ORCA-IN
**Dream Team**	Metastatic Prostate Cancer	266	(17)
**MBC Project**	Metastatic Breast Cancer	146	http://www.cbioportal.org/study/summary?id=brca_mbcproject_wagle_2017
**TCGA-SKCM-MET**	Metastatic Melanoma	368	(18)
		**1,541**	

Table S3. Top 200 genes identified by RF learning being most discriminative for CXCR4 high vs. CXCR4 low expressing cancer samples.

ENSG Number	HGNC	Description
ENSG00000121966	CXCR4	C-X-C motif chemokine receptor 4 [Source:HGNC Symbol;Acc:HGNC:2561]
ENSG00000169508	GPR183	G protein-coupled receptor 183 [Source:HGNC Symbol;Acc:HGNC:3128]
ENSG00000115165	CYTIP	cytohesin 1 interacting protein [Source:HGNC Symbol;Acc:HGNC:9506]
ENSG00000090104	RGS1	regulator of G protein signaling 1 [Source:HGNC Symbol;Acc:HGNC:9991]
ENSG00000110848	CD69	CD69 molecule [Source:HGNC Symbol;Acc:HGNC:1694]
ENSG00000224137	LINC01857	long intergenic non-protein coding RNA 1857 [Source:HGNC Symbol;Acc:HGNC:52673]
ENSG00000105369	CD79A	CD79a molecule [Source:HGNC Symbol;Acc:HGNC:1698]
ENSG00000102245	CD40LG	CD40 ligand [Source:HGNC Symbol;Acc:HGNC:11935]
ENSG00000255760	LINC02422	long intergenic non-protein coding RNA 2422 [Source:HGNC Symbol;Acc:HGNC:53352]
ENSG00000104894	CD37	CD37 molecule [Source:HGNC Symbol;Acc:HGNC:1666]
ENSG00000240505	TNFRSF13B	TNF receptor superfamily member 13B [Source:HGNC Symbol;Acc:HGNC:18153]
ENSG00000136573	BLK	BLK proto-oncogene, Src family tyrosine kinase [Source:HGNC Symbol;Acc:HGNC:1057]
ENSG00000177455	CD19	CD19 molecule [Source:HGNC Symbol;Acc:HGNC:1633]
ENSG00000084070	SMAP2	small ArfGAP2 [Source:HGNC Symbol;Acc:HGNC:25082]
ENSG00000156738	MS4A1	membrane spanning 4-domains A1 [Source:HGNC Symbol;Acc:HGNC:7315]
ENSG00000269404	SPIB	Spi-B transcription factor [Source:HGNC Symbol;Acc:HGNC:11242]
ENSG00000117322	CR2	complement C3d receptor 2 [Source:HGNC Symbol;Acc:HGNC:2336]
ENSG00000279024		
ENSG00000159958	TNFRSF13C	TNF receptor superfamily member 13C [Source:HGNC Symbol;Acc:HGNC:17755]
ENSG00000172724	CCL19	C-C motif chemokine ligand 19 [Source:HGNC Symbol;Acc:HGNC:10617]
ENSG00000274961		
ENSG00000128218	VPREB3	V-set pre-B cell surrogate light chain 3 [Source:HGNC Symbol;Acc:HGNC:12710]
ENSG00000111796	KLRB1	killer cell lectin like receptor B1 [Source:HGNC Symbol;Acc:HGNC:6373]
ENSG00000126353	CCR7	C-C motif chemokine receptor 7 [Source:HGNC Symbol;Acc:HGNC:1608]

ENSG00000105366	SIGLEC8	sialic acid binding Ig like lectin 8 [Source:HGNC Symbol;Acc:HGNC:10877]
ENSG00000250850	AL161781.2	novel transcript, antisense to PAX5
ENSG00000267583	AC007998.3	novel transcript
ENSG00000247774	PCED1B-AS1	PCED1B antisense RNA 1 [Source:HGNC Symbol;Acc:HGNC:44166]
ENSG00000162739	SLAMF6	SLAM family member 6 [Source:HGNC Symbol;Acc:HGNC:21392]
ENSG00000117091	CD48	CD48 molecule [Source:HGNC Symbol;Acc:HGNC:1683]
ENSG00000079263	SP140	SP140 nuclear body protein [Source:HGNC Symbol;Acc:HGNC:17133]
ENSG00000235304	LINC01281	long intergenic non-protein coding RNA 1281 [Source:HGNC Symbol;Acc:HGNC:50337]
ENSG00000187912	CLEC17A	C-type lectin domain containing 17A [Source:HGNC Symbol;Acc:HGNC:34520]
ENSG00000122224	LY9	lymphocyte antigen 9 [Source:HGNC Symbol;Acc:HGNC:6730]
ENSG00000154016	GRAP	GRB2 related adaptor protein [Source:HGNC Symbol;Acc:HGNC:4562]
ENSG00000128917	DLL4	delta like canonical Notch ligand 4 [Source:HGNC Symbol;Acc:HGNC:2910]
ENSG00000241106	HLA-DOB	major histocompatibility complex, class II, DO beta [Source:HGNC Symbol;Acc:HGNC:4937]
ENSG00000160683	CXCR5	C-X-C motif chemokine receptor 5 [Source:HGNC Symbol;Acc:HGNC:1060]
ENSG00000162894	FCMR	Fc fragment of IgM receptor [Source:HGNC Symbol;Acc:HGNC:14315]
ENSG00000186265	BTLA	B and T lymphocyte associated [Source:HGNC Symbol;Acc:HGNC:21087]
ENSG00000156234	CXCL13	C-X-C motif chemokine ligand 13 [Source:HGNC Symbol;Acc:HGNC:10639]
ENSG00000171388	APLN	apelin [Source:HGNC Symbol;Acc:HGNC:16665]
ENSG00000131401	NAPSB	napsin B aspartic peptidase, pseudogene [Source:HGNC Symbol;Acc:HGNC:13396]
ENSG00000277030	MIR8071-2	microRNA 8071-2 [Source:HGNC Symbol;Acc:HGNC:49958]
ENSG00000258572	AL133467.1	novel transcript
ENSG00000167483	FAM129C	family with sequence similarity 129 member C [Source:HGNC Symbol;Acc:HGNC:24130]
ENSG00000007312	CD79B	CD79b molecule [Source:HGNC Symbol;Acc:HGNC:1699]
ENSG00000161940	BCL6B	BCL6B, transcription repressor [Source:HGNC Symbol;Acc:HGNC:1002]
ENSG00000106952	TNFSF8	TNF superfamily member 8 [Source:HGNC Symbol;Acc:HGNC:11938]
ENSG00000181847	TIGIT	T cell immunoreceptor with Ig and ITIM domains [Source:HGNC Symbol;Acc:HGNC:26838]
ENSG00000155926	SLA	Src like adaptor [Source:HGNC Symbol;Acc:HGNC:10902]
ENSG00000066056	TIE1	tyrosine kinase with immunoglobulin like and EGF like domains 1 [Source:HGNC Symbol;Acc:HGNC:11809]
ENSG00000128438	TBC1D27P	TBC1 domain family member 27, pseudogene [Source:HGNC Symbol;Acc:HGNC:28104]
ENSG00000160856	FCRL3	Fc receptor like 3 [Source:HGNC Symbol;Acc:HGNC:18506]
ENSG00000277054		
ENSG00000110777	POU2AF1	POU class 2 associating factor 1 [Source:HGNC Symbol;Acc:HGNC:9211]
ENSG00000226979	LTA	lymphotoxin alpha [Source:HGNC Symbol;Acc:HGNC:6709]
ENSG00000078589	P2RY10	P2Y receptor family member 10 [Source:HGNC Symbol;Acc:HGNC:19906]
ENSG00000139193	CD27	CD27 molecule [Source:HGNC Symbol;Acc:HGNC:11922]
ENSG00000183813	CCR4	C-C motif chemokine receptor 4 [Source:HGNC Symbol;Acc:HGNC:1605]
ENSG00000113555	PCDH12	protocadherin 12 [Source:HGNC Symbol;Acc:HGNC:8657]
ENSG00000163534	FCRL1	Fc receptor like 1 [Source:HGNC Symbol;Acc:HGNC:18509]
ENSG00000109471	IL2	interleukin 2 [Source:HGNC Symbol;Acc:HGNC:6001]
ENSG00000163599	CTLA4	cytotoxic T-lymphocyte associated protein 4 [Source:HGNC Symbol;Acc:HGNC:2505]
ENSG00000197405	C5AR1	complement C5a receptor 1 [Source:HGNC Symbol;Acc:HGNC:1338]
ENSG00000265929	MIR5195	microRNA 5195 [Source:HGNC Symbol;Acc:HGNC:43526]
ENSG00000211734	TRBV5-1	T cell receptor beta variable 5-1 [Source:HGNC Symbol;Acc:HGNC:12218]

ENSG00000028137	TNFRSF1B	TNF receptor superfamily member 1B [Source:HGNC Symbol;Acc:HGNC:11917]
ENSG00000137101	CD72	CD72 molecule [Source:HGNC Symbol;Acc:HGNC:1696]
ENSG00000113263	ITK	IL2 inducible T cell kinase [Source:HGNC Symbol;Acc:HGNC:6171]
ENSG00000133639	BTG1	BTG anti-proliferation factor 1 [Source:HGNC Symbol;Acc:HGNC:1130]
ENSG00000124203	ZNF831	zinc finger protein 831 [Source:HGNC Symbol;Acc:HGNC:16167]
ENSG00000253701		
ENSG00000118503	TNFAIP3	TNF alpha induced protein 3 [Source:HGNC Symbol;Acc:HGNC:11896]
ENSG00000147113	DIPK2B	divergent protein kinase domain 2B [Source:HGNC Symbol;Acc:HGNC:25866]
ENSG00000075884	ARHGAP15	Rho GTPase activating protein 15 [Source:HGNC Symbol;Acc:HGNC:21030]
ENSG00000205056	LINC02397	long intergenic non-protein coding RNA 2397 [Source:HGNC Symbol;Acc:HGNC:53323]
ENSG00000185862	EVI2B	ecotropic viral integration site 2B [Source:HGNC Symbol;Acc:HGNC:3500]
ENSG00000013725	CD6	CD6 molecule [Source:HGNC Symbol;Acc:HGNC:1691]
ENSG00000235532	LINC00402	long intergenic non-protein coding RNA 402 [Source:HGNC Symbol;Acc:HGNC:42732]
ENSG00000189152	GRAPL	GRB2 related adaptor protein like [Source:HGNC Symbol;Acc:HGNC:37240]
ENSG00000271680	AC098935.2	La ribonucleoprotein domain family, member 1B (LARP1B) pseudogene
ENSG00000110448	CD5	CD5 molecule [Source:HGNC Symbol;Acc:HGNC:1685]
ENSG00000104921	FCER2	Fc fragment of IgE receptor II [Source:HGNC Symbol;Acc:HGNC:3612]
ENSG00000168685	IL7R	interleukin 7 receptor [Source:HGNC Symbol;Acc:HGNC:6024]
ENSG00000198771	RCSD1	RCSD domain containing 1 [Source:HGNC Symbol;Acc:HGNC:28310]
ENSG00000143119	CD53	CD53 molecule [Source:HGNC Symbol;Acc:HGNC:1686]
ENSG00000257275	AL139020.2	novel transcript, antisense to TCL1A
ENSG00000203859	HSD3B2	hydroxy-delta-5-steroid dehydrogenase, 3 beta- and steroid delta-isomerase 2 [Source:HGNC Symbol;Acc:HGNC:5218]
ENSG00000196092	PAX5	paired box 5 [Source:HGNC Symbol;Acc:HGNC:8619]
ENSG00000163219	ARHGAP25	Rho GTPase activating protein 25 [Source:HGNC Symbol;Acc:HGNC:28951]
ENSG00000254029	IGLC4	immunoglobulin lambda constant 4 (pseudogene) [Source:HGNC Symbol;Acc:HGNC:5858]
ENSG00000100721	AL139020.1	T cell leukemia/lymphoma 1A [Source:NCBI gene;Acc:8115]
ENSG00000236800	AC068898.1	novel transcript
ENSG00000241490	AC093010.2	novel transcript, antisense to ZNF80
ENSG00000247982	LINC00926	long intergenic non-protein coding RNA 926 [Source:HGNC Symbol;Acc:HGNC:27514]
ENSG00000178567	EPM2AIP1	EPM2A interacting protein 1 [Source:HGNC Symbol;Acc:HGNC:19735]
ENSG00000174946	GPR171	G protein-coupled receptor 171 [Source:HGNC Symbol;Acc:HGNC:30057]
ENSG00000113088	GZMK	granzyme K [Source:HGNC Symbol;Acc:HGNC:4711]
ENSG00000184293	CLECL1	C-type lectin like 1 [Source:HGNC Symbol;Acc:HGNC:24462]
ENSG00000179776	CDH5	cadherin 5 [Source:HGNC Symbol;Acc:HGNC:1764]
ENSG00000255354		
ENSG00000164691	TAGAP	T cell activation RhoGTPase activating protein [Source:HGNC Symbol;Acc:HGNC:15669]
ENSG00000272870	AC097534.2	novel transcript, antisense to SAP30
ENSG00000178562	CD28	CD28 molecule [Source:HGNC Symbol;Acc:HGNC:1653]
ENSG00000110324	IL10RA	interleukin 10 receptor subunit alpha [Source:HGNC Symbol;Acc:HGNC:5964]
ENSG00000253140	AC026904.1	novel transcript
ENSG00000239961	LILRA4	leukocyte immunoglobulin like receptor A4 [Source:HGNC Symbol;Acc:HGNC:15503]
ENSG00000169442	CD52	CD52 molecule [Source:HGNC Symbol;Acc:HGNC:1804]
ENSG00000132854	KANK4	KN motif and ankyrin repeat domains 4 [Source:HGNC Symbol;Acc:HGNC:27263]

ENSG00000198851	CD3E	CD3e molecule [Source:HGNC Symbol;Acc:HGNC:1674]
ENSG00000236213	AC006369.1	novel transcript
ENSG00000076242	MLH1	mutL homolog 1 [Source:HGNC Symbol;Acc:HGNC:7127]
ENSG00000132704	FCRL2	Fc receptor like 2 [Source:HGNC Symbol;Acc:HGNC:14875]
ENSG00000182866	LCK	LCK proto-oncogene, Src family tyrosine kinase [Source:HGNC Symbol;Acc:HGNC:6524]
ENSG00000157514	TSC22D3	TSC22 domain family member 3 [Source:HGNC Symbol;Acc:HGNC:3051]
ENSG00000232869	TRBV29-1	T cell receptor beta variable 29-1 [Source:HGNC Symbol;Acc:HGNC:12210]
ENSG00000180096	Sep 01	septin 1 [Source:HGNC Symbol;Acc:HGNC:2879]
ENSG00000015285	WAS	Wiskott-Aldrich syndrome [Source:HGNC Symbol;Acc:HGNC:12731]
ENSG00000204475	NCR3	natural cytotoxicity triggering receptor 3 [Source:HGNC Symbol;Acc:HGNC:19077]
ENSG00000211706	TRBV6-1	T cell receptor beta variable 6-1 [Source:HGNC Symbol;Acc:HGNC:12226]
ENSG00000275418	AC104971.3	novel transcript
ENSG00000253364	AL928742.1	novel transcript
ENSG00000198821	CD247	CD247 molecule [Source:HGNC Symbol;Acc:HGNC:1677]
ENSG00000211772	TRBC2	T cell receptor beta constant 2 [Source:HGNC Symbol;Acc:HGNC:12157]
ENSG00000172578	KLHL6	kelch like family member 6 [Source:HGNC Symbol;Acc:HGNC:18653]
ENSG00000253451	IGLV2-28	immunoglobulin lambda variable 2-28 (pseudogene) [Source:HGNC Symbol;Acc:HGNC:5891]
ENSG00000103522	IL21R	interleukin 21 receptor [Source:HGNC Symbol;Acc:HGNC:6006]
ENSG00000162951	LRRTM1	leucine rich repeat transmembrane neuronal 1 [Source:HGNC Symbol;Acc:HGNC:19408]
ENSG00000254030	IGLC5	immunoglobulin lambda constant 5 (pseudogene) [Source:HGNC Symbol;Acc:HGNC:5859]
ENSG00000163519	TRAT1	T cell receptor associated transmembrane adaptor 1 [Source:HGNC Symbol;Acc:HGNC:30698]
ENSG00000007908	SELE	selectin E [Source:HGNC Symbol;Acc:HGNC:10718]
ENSG00000158050	DUSP2	dual specificity phosphatase 2 [Source:HGNC Symbol;Acc:HGNC:3068]
ENSG00000100100	PIK3IP1	phosphoinositide-3-kinase interacting protein 1 [Source:HGNC Symbol;Acc:HGNC:24942]
ENSG00000261371	PECAM1	platelet and endothelial cell adhesion molecule 1 [Source:HGNC Symbol;Acc:HGNC:8823]
ENSG00000185669	SNAI3	snail family transcriptional repressor 3 [Source:NCBI gene;Acc:333929]
ENSG00000141506	PIK3R5	phosphoinositide-3-kinase regulatory subunit 5 [Source:HGNC Symbol;Acc:HGNC:30035]
ENSG00000143149	ALDH9A1	aldehyde dehydrogenase 9 family member A1 [Source:HGNC Symbol;Acc:HGNC:412]
ENSG00000107672	NSMCE4A	NSE4 homolog A, SMC5-SMC6 complex component [Source:HGNC Symbol;Acc:HGNC:25935]
ENSG00000104856	RELB	RELB proto-oncogene, NF-kB subunit [Source:HGNC Symbol;Acc:HGNC:9956]
ENSG00000164105	SAP30	Sin3A associated protein 30 [Source:HGNC Symbol;Acc:HGNC:10532]
ENSG00000142512	SIGLEC10	sialic acid binding Ig like lectin 10 [Source:HGNC Symbol;Acc:HGNC:15620]
ENSG00000105639	JAK3	Janus kinase 3 [Source:HGNC Symbol;Acc:HGNC:6193]
ENSG00000237513	AC007384.1	novel transcript
ENSG00000166199	ALKBH3	alkB homolog 3, alpha-ketoglutaratedependent dioxygenase [Source:HGNC Symbol;Acc:HGNC:30141]
ENSG00000174123	TLR10	toll like receptor 10 [Source:HGNC Symbol;Acc:HGNC:15634]
ENSG00000270164	LINC01480	long intergenic non-protein coding RNA 1480 [Source:HGNC Symbol;Acc:HGNC:51124]
ENSG00000260303	AC108206.1	novel transcript
ENSG00000227507	LTB	lymphotoxin beta [Source:HGNC Symbol;Acc:HGNC:6711]
ENSG00000276557	TRBV18	T cell receptor beta variable 18 [Source:HGNC Symbol;Acc:HGNC:12193]

ENSG00000207835		
ENSG00000174175	SELP	selectin P [Source:HGNC Symbol;Acc:HGNC:10721]
ENSG00000083307	GRHL2	grainyhead like transcription factor 2 [Source:HGNC Symbol;Acc:HGNC:2799]
ENSG00000230709	AC104024.1	novel transcript
ENSG00000120129	DUSP1	dual specificity phosphatase 1 [Source:HGNC Symbol;Acc:HGNC:3064]
ENSG00000268027	AC243960.1	novel transcript, sense intronic to CEACAM21
ENSG00000135048	CEMIP2	cell migration inducing hyaluronidase 2 [Source:HGNC Symbol;Acc:HGNC:11869]
ENSG00000132906	CASP9	caspase 9 [Source:HGNC Symbol;Acc:HGNC:1511]
ENSG00000171532	NEUROD2	neuronal differentiation 2 [Source:HGNC Symbol;Acc:HGNC:7763]
ENSG00000234965	SHISA8	shisa family member 8 [Source:HGNC Symbol;Acc:HGNC:18351]
ENSG00000273837	AC018755.4	novel transcript, sense intronic to AC018755.18
ENSG00000237702	TRBV3-1	T cell receptor beta variable 3-1 [Source:HGNC Symbol;Acc:HGNC:12212]
ENSG00000163518	FCRL4	Fc receptor like 4 [Source:HGNC Symbol;Acc:HGNC:18507]
ENSG00000162511	LAPTM5	lysosomal protein transmembrane 5 [Source:HGNC Symbol;Acc:HGNC:29612]
ENSG00000117090	SLAMF1	signaling lymphocytic activation molecule family member 1 [Source:HGNC Symbol;Acc:HGNC:10903]
ENSG00000251126	AC017007.5	novel transcript, antisense to ANK2
ENSG00000253234	IGLV2-5	immunoglobulin lambda variable 2-5 (pseudogene) [Source:HGNC Symbol;Acc:HGNC:5894]
ENSG00000225098	BCRP1	breakpoint cluster region pseudogene 1 [Source:HGNC Symbol;Acc:HGNC:39073]
ENSG00000082556	OPRK1	opioid receptor kappa 1 [Source:HGNC Symbol;Acc:HGNC:8154]
ENSG00000100368	CSF2RB	colony stimulating factor 2 receptor beta common subunit [Source:HGNC Symbol;Acc:HGNC:2436]
ENSG00000181617	FDCSP	follicular dendritic cell secreted protein [Source:HGNC Symbol;Acc:HGNC:19215]
ENSG00000153064	BANK1	B cell scaffold protein with ankyrin repeats 1 [Source:HGNC Symbol;Acc:HGNC:18233]
ENSG00000174255	ZNF80	zinc finger protein 80 [Source:HGNC Symbol;Acc:HGNC:13155]
ENSG00000146192	FGD2	FYVE, RhoGEF and PH domain containing 2 [Source:HGNC Symbol;Acc:HGNC:3664]
ENSG00000246582	AC100861.1	novel transcript, antisense to TNFRSF10A
ENSG00000254667		
ENSG00000250208	FZD10-AS1	FZD10 antisense divergent transcript [Source:HGNC Symbol;Acc:HGNC:48632]
ENSG00000049768	FOXP3	forkhead box P3 [Source:HGNC Symbol;Acc:HGNC:6106]
ENSG00000105246	EBI3	Epstein-Barr virus induced 3 [Source:HGNC Symbol;Acc:HGNC:3129]
ENSG00000182518	FAM104B	family with sequence similarity 104 member B [Source:HGNC Symbol;Acc:HGNC:25085]
ENSG00000128815	WDFY4	WDFY family member 4 [Source:HGNC Symbol;Acc:HGNC:29323]
ENSG00000211746	TRBV19	T cell receptor beta variable 19 [Source:HGNC Symbol;Acc:HGNC:12194]
ENSG00000225391	AL135902.1	novel transcript
ENSG00000275801	AL121985.3	SLAM family member 9 (SLAMF9) pseudogene
ENSG00000274172	MIR8071-1	microRNA 8071-1 [Source:HGNC Symbol;Acc:HGNC:50041]
ENSG00000265517		
ENSG00000162461	SLC25A34	solute carrier family 25 member 34 [Source:HGNC Symbol;Acc:HGNC:27653]
ENSG00000159723	AGRP	agouti related neuropeptide [Source:HGNC Symbol;Acc:HGNC:330]
ENSG00000166265	CYYR1	cysteine and tyrosine rich 1 [Source:HGNC Symbol;Acc:HGNC:16274]
ENSG00000271046	AL512631.1	novel transcript
ENSG00000262488	AC133065.2	Rho GTPase activating protein 21 (ARHGAP21) pseudogene
ENSG00000214376	VSTM5	V-set and transmembrane domain containing 5 [Source:HGNC Symbol;Acc:HGNC:34443]

ENSG00000131386	GALNT15	polypeptide N-acetylgalactosaminyltransferase 15 [Source:HGNC Symbol;Acc:HGNC:21531]
ENSG00000233093	LINC00892	long intergenic non-protein coding RNA 892 [Source:HGNC Symbol;Acc:HGNC:48578]
ENSG00000123342	MMP19	matrix metallopeptidase 19 [Source:HGNC Symbol;Acc:HGNC:7165]
ENSG00000122986	HVCN1	hydrogen voltage gated channel 1 [Source:HGNC Symbol;Acc:HGNC:28240]
ENSG00000188822	CNR2	cannabinoid receptor 2 [Source:HGNC Symbol;Acc:HGNC:2160]
ENSG00000140968	IRF8	interferon regulatory factor 8 [Source:HGNC Symbol;Acc:HGNC:5358]
ENSG00000010671	BTK	Bruton tyrosine kinase [Source:HGNC Symbol;Acc:HGNC:1133]
ENSG00000211753	TRBV28	T cell receptor beta variable 28 [Source:HGNC Symbol;Acc:HGNC:12209]

Table S4. Top 200 genes identified by RF learning being most discriminative for FAP high vs. FAP low expressing cancer samples.

ENSG Number	HGNC	Description
ENSG00000078098	FAP	fibroblast activation protein alpha [Source:HGNC Symbol;Acc:HGNC:3590]
ENSG00000164932	CTHRC1	collagen triple helix repeat containing 1 [Source:HGNC Symbol;Acc:HGNC:18831]
ENSG00000151388	ADAMTS12	ADAM metallopeptidase with thrombospondin type 1 motif 12 [Source:HGNC Symbol;Acc:HGNC:14605]
ENSG00000115414	FN1	fibronectin 1 [Source:HGNC Symbol;Acc:HGNC:3778]
ENSG00000120820	GLT8D2	glycosyltransferase 8 domain containing 2 [Source:HGNC Symbol;Acc:HGNC:24890]
ENSG00000108821	COL1A1	collagen type I alpha 1 chain [Source:HGNC Symbol;Acc:HGNC:2197]
ENSG00000169604	ANTXR1	ANTXR cell adhesion molecule 1 [Source:HGNC Symbol;Acc:HGNC:21014]
ENSG00000236841	AC007750.1	novel transcript
ENSG00000204262	COL5A2	collagen type V alpha 2 chain [Source:HGNC Symbol;Acc:HGNC:2210]
ENSG00000137573	SULF1	sulfatase 1 [Source:HGNC Symbol;Acc:HGNC:20391]
ENSG00000130635	COL5A1	collagen type V alpha 1 chain [Source:HGNC Symbol;Acc:HGNC:2209]
ENSG00000163359	COL6A3	collagen type VI alpha 3 chain [Source:HGNC Symbol;Acc:HGNC:2213]
ENSG00000148848	ADAM12	ADAM metallopeptidase domain 12 [Source:HGNC Symbol;Acc:HGNC:190]
ENSG00000164692	COL1A2	collagen type I alpha 2 chain [Source:HGNC Symbol;Acc:HGNC:2198]
ENSG00000122641	INHBA	inhibin subunit beta A [Source:HGNC Symbol;Acc:HGNC:6066]
ENSG00000186340	THBS2	thrombospondin 2 [Source:HGNC Symbol;Acc:HGNC:11786]
ENSG00000132000	PODNL1	podocan like 1 [Source:HGNC Symbol;Acc:HGNC:26275]
ENSG00000087245	MMP2	matrix metallopeptidase 2 [Source:HGNC Symbol;Acc:HGNC:7166]
ENSG00000168542	COL3A1	collagen type III alpha 1 chain [Source:HGNC Symbol;Acc:HGNC:2201]
ENSG00000230838	LINC01614	long intergenic non-protein coding RNA 1614 [Source:HGNC Symbol;Acc:HGNC:51847]
ENSG00000182492	BGN	biglycan [Source:HGNC Symbol;Acc:HGNC:1044]
ENSG00000139329	LUM	lumican [Source:HGNC Symbol;Acc:HGNC:6724]
ENSG00000133110	POSTN	periostin [Source:HGNC Symbol;Acc:HGNC:16953]
ENSG00000162745	OLFML2B	olfactomedin like 2B [Source:HGNC Symbol;Acc:HGNC:24558]
ENSG00000101825	MXRA5	matrix remodeling associated 5 [Source:HGNC Symbol;Acc:HGNC:7539]
ENSG00000140937	CDH11	cadherin 11 [Source:HGNC Symbol;Acc:HGNC:1750]
ENSG00000267013	LINC01929	long intergenic non-protein coding RNA 1929 [Source:HGNC Symbol;Acc:HGNC:52751]
ENSG00000113140	SPARC	secreted protein acidic and cysteine rich [Source:HGNC Symbol;Acc:HGNC:11219]
ENSG00000113083	LOX	lysyl oxidase [Source:HGNC Symbol;Acc:HGNC:6664]

ENSG00000122786	CALD1	caldesmon 1 [Source:HGNC Symbol;Acc:HGNC:1441]
ENSG00000106624	AEBP1	AE binding protein 1 [Source:HGNC Symbol;Acc:HGNC:303]
ENSG00000174325	DIRC1	disrupted in renal carcinoma 1 [Source:HGNC Symbol;Acc:HGNC:15760]
ENSG00000279881		
ENSG00000087116	ADAMTS2	ADAM metallopeptidase with thrombospondin type 1 motif 2 [Source:HGNC Symbol;Acc:HGNC:218]
ENSG00000167123	CERCAM	cerebral endothelial cell adhesion molecule [Source:HGNC Symbol;Acc:HGNC:23723]
ENSG00000180044	C3orf80	chromosome 3 open reading frame 80 [Source:HGNC Symbol;Acc:HGNC:40048]
ENSG00000134013	LOXL2	lysyl oxidase like 2 [Source:HGNC Symbol;Acc:HGNC:6666]
ENSG00000137809	ITGA11	integrin subunit alpha 11 [Source:HGNC Symbol;Acc:HGNC:6136]
ENSG00000083782	EPYC	epiphycan [Source:HGNC Symbol;Acc:HGNC:3053]
ENSG00000144810	COL8A1	collagen type VIII alpha 1 chain [Source:HGNC Symbol;Acc:HGNC:2215]
ENSG00000060718	COL11A1	collagen type XI alpha 1 chain [Source:HGNC Symbol;Acc:HGNC:2186]
ENSG00000111799	COL12A1	collagen type XII alpha 1 chain [Source:HGNC Symbol;Acc:HGNC:2188]
ENSG00000263655	AC090125.1	novel transcript
ENSG00000149380	P4HA3	prolyl 4-hydroxylase subunit alpha 3 [Source:HGNC Symbol;Acc:HGNC:30135]
ENSG00000154096	THY1	Thy-1 cell surface antigen [Source:HGNC Symbol;Acc:HGNC:11801]
ENSG00000124813	RUNX2	runt related transcription factor 2 [Source:HGNC Symbol;Acc:HGNC:10472]
ENSG00000123500	COL10A1	collagen type X alpha 1 chain [Source:HGNC Symbol;Acc:HGNC:2185]
ENSG00000104415	CCN4	cellular communication network factor 4 [Source:HGNC Symbol;Acc:HGNC:12769]
ENSG00000138316	ADAMTS14	ADAM metallopeptidase with thrombospondin type 1 motif 14 [Source:HGNC Symbol;Acc:HGNC:14899]
ENSG00000123610	TNFAIP6	TNF alpha induced protein 6 [Source:HGNC Symbol;Acc:HGNC:11898]
ENSG00000106483	SFRP4	secreted frizzled related protein 4 [Source:HGNC Symbol;Acc:HGNC:10778]
ENSG00000157227	MMP14	matrix metallopeptidase 14 [Source:HGNC Symbol;Acc:HGNC:7160]
ENSG00000106333	PCOLCE	procollagen C-endopeptidase enhancer [Source:HGNC Symbol;Acc:HGNC:8738]
ENSG00000244564	AC096888.1	novel transcript
ENSG00000122861	PLAU	plasminogen activator, urokinase [Source:HGNC Symbol;Acc:HGNC:9052]
ENSG00000227964	LINC01429	long intergenic non-protein coding RNA 1429 [Source:HGNC Symbol;Acc:HGNC:50741]
ENSG00000079150	FKBP7	FKBP prolyl isomerase 7 [Source:HGNC Symbol;Acc:HGNC:3723]
ENSG00000133466	C1QTNF6	C1q and TNF related 6 [Source:HGNC Symbol;Acc:HGNC:14343]
ENSG00000106819	ASPN	asporin [Source:HGNC Symbol;Acc:HGNC:14872]
ENSG00000142173	COL6A2	collagen type VI alpha 2 chain [Source:HGNC Symbol;Acc:HGNC:2212]
ENSG00000261327	AC134312.5	novel transcript
ENSG00000113721	PDGFRB	platelet derived growth factor receptor beta [Source:HGNC Symbol;Acc:HGNC:8804]
ENSG00000143387	CTSK	cathepsin K [Source:HGNC Symbol;Acc:HGNC:2536]
ENSG00000165617	DACT1	dishevelled binding antagonist of beta catenin 1 [Source:HGNC Symbol;Acc:HGNC:17748]
ENSG00000203740	METTL11B	methyltransferase like 11B [Source:HGNC Symbol;Acc:HGNC:31932]
ENSG00000120708	TGFBI	transforming growth factor beta induced [Source:HGNC Symbol;Acc:HGNC:11771]
ENSG00000123342	MMP19	matrix metallopeptidase 19 [Source:HGNC Symbol;Acc:HGNC:7165]
ENSG00000164294	GPX8	glutathione peroxidase 8 (putative) [Source:HGNC Symbol;Acc:HGNC:33100]
ENSG00000203805	PLPP4	phospholipid phosphatase 4 [Source:HGNC Symbol;Acc:HGNC:23531]
ENSG00000243742	RPLP0P2	ribosomal protein lateral stalk subunit P0 pseudogene 2 [Source:HGNC Symbol;Acc:HGNC:17960]
ENSG00000011465	DCN	decorin [Source:HGNC Symbol;Acc:HGNC:2705]
ENSG00000116132	PRRX1	paired related homeobox 1 [Source:HGNC Symbol;Acc:HGNC:9142]

ENSG00000222032	AC112721.2	novel protein (LOC728009)
ENSG00000261295		
ENSG00000230333	AC004160.1	novel transcript, antisense to THSD7A
ENSG00000074590	NUAK1	NUAK family kinase 1 [Source:HGNC Symbol;Acc:HGNC:14311]
ENSG00000227496	AC099066.2	novel transcript
ENSG00000168487	BMP1	bone morphogenetic protein 1 [Source:HGNC Symbol;Acc:HGNC:1067]
ENSG00000223485	LINC01615	long intergenic non-protein coding RNA 1615 [Source:HGNC Symbol;Acc:HGNC:51898]
ENSG00000178033	CALHM5	calcium homeostasis modulator family member 5 [Source:HGNC Symbol;Acc:HGNC:21568]
ENSG00000142156	COL6A1	collagen type VI alpha 1 chain [Source:HGNC Symbol;Acc:HGNC:2211]
ENSG00000166250	CLMP	CXADR like membrane protein [Source:HGNC Symbol;Acc:HGNC:24039]
ENSG00000142552	RCN3	reticulocalbin 3 [Source:HGNC Symbol;Acc:HGNC:21145]
ENSG00000080573	COL5A3	collagen type V alpha 3 chain [Source:HGNC Symbol;Acc:HGNC:14864]
ENSG00000102359	SRPX2	sushi repeat containing protein X-linked 2 [Source:HGNC Symbol;Acc:HGNC:30668]
ENSG00000038427	VCAN	versican [Source:HGNC Symbol;Acc:HGNC:2464]
ENSG00000140682	TGFB1I1	transforming growth factor beta 1 induced transcript 1 [Source:HGNC Symbol;Acc:HGNC:11767]
ENSG00000119681	LTBP2	latent transforming growth factor beta binding protein 2 [Source:HGNC Symbol;Acc:HGNC:6715]
ENSG00000163430	FSTL1	follistatin like 1 [Source:HGNC Symbol;Acc:HGNC:3972]
ENSG00000225614	ZNF469	zinc finger protein 469 [Source:HGNC Symbol;Acc:HGNC:23216]
ENSG00000166147	FBN1	fibrillin 1 [Source:NCBI gene;Acc:2200]
ENSG00000198108	CHSY3	chondroitin sulfate synthase 3 [Source:HGNC Symbol;Acc:HGNC:24293]
ENSG00000222022	AC112721.1	novel transcript
ENSG00000172061	LRRC15	leucine rich repeat containing 15 [Source:HGNC Symbol;Acc:HGNC:20818]
ENSG00000086991	NOX4	NADPH oxidase 4 [Source:HGNC Symbol;Acc:HGNC:7891]
ENSG00000229720	AL109924.2	novel transcript
ENSG00000106366	SERPINE1	serpin family E member 1 [Source:HGNC Symbol;Acc:HGNC:8583]
ENSG00000099953	MMP11	matrix metallopeptidase 11 [Source:HGNC Symbol;Acc:HGNC:7157]
ENSG00000102970	CCL17	C-C motif chemokine ligand 17 [Source:HGNC Symbol;Acc:HGNC:10615]
ENSG00000231123	SPATA20P1	spermatogenesis associated 20 pseudogene 1 [Source:HGNC Symbol;Acc:HGNC:39636]
ENSG00000138675	FGF5	fibroblast growth factor 5 [Source:HGNC Symbol;Acc:HGNC:3683]
ENSG00000137745	MMP13	matrix metallopeptidase 13 [Source:HGNC Symbol;Acc:HGNC:7159]
ENSG00000102802	MEDAG	mesenteric estrogen dependent adipogenesis [Source:HGNC Symbol;Acc:HGNC:25926]
ENSG00000149591	TAGLN	transgelin [Source:HGNC Symbol;Acc:HGNC:11553]
ENSG00000149257	SERPINH1	serpin family H member 1 [Source:HGNC Symbol;Acc:HGNC:1546]
ENSG00000259807	AC009093.1	novel transcript
ENSG00000183801	OLFML1	olfactomedin like 1 [Source:HGNC Symbol;Acc:HGNC:24473]
ENSG00000128595	CALU	calumenin [Source:HGNC Symbol;Acc:HGNC:1458]
ENSG00000129009	ISLR	immunoglobulin superfamily containing leucine rich repeat [Source:HGNC Symbol;Acc:HGNC:6133]
ENSG00000232679	LINC01705	long intergenic non-protein coding RNA 1705 [Source:HGNC Symbol;Acc:HGNC:52493]
ENSG00000233521	LINC01638	long intergenic non-protein coding RNA 1638 [Source:HGNC Symbol;Acc:HGNC:52425]
ENSG00000162493	PDPN	podoplanin [Source:HGNC Symbol;Acc:HGNC:29602]
ENSG00000117586	TNFSF4	TNF superfamily member 4 [Source:HGNC Symbol;Acc:HGNC:11934]
ENSG00000256235	SMIM3	small integral membrane protein 3 [Source:HGNC Symbol;Acc:HGNC:30248]
ENSG00000117385	P3H1	prolyl 3-hydroxylase 1 [Source:HGNC Symbol;Acc:HGNC:19316]

ENSG00000105472	CLEC11A	C-type lectin domain containing 11A [Source:HGNC Symbol;Acc:HGNC:10576]
ENSG00000198542	ITGBL1	integrin subunit beta like 1 [Source:HGNC Symbol;Acc:HGNC:6164]
ENSG00000011028	AC080038.1	mannose receptor C type 2 [Source:NCBI gene;Acc:9902]
ENSG00000035862	TIMP2	TIMP metallopeptidase inhibitor 2 [Source:HGNC Symbol;Acc:HGNC:11821]
ENSG00000131459	GFPT2	glutamine-fructose-6-phosphate transaminase 2 [Source:HGNC Symbol;Acc:HGNC:4242]
ENSG00000087303	NID2	nidogen 2 [Source:HGNC Symbol;Acc:HGNC:13389]
ENSG00000278902		
ENSG00000164694	FNDC1	fibronectin type III domain containing 1 [Source:HGNC Symbol;Acc:HGNC:21184]
ENSG00000179300	RTL3	retrotransposon Gag like 3 [Source:HGNC Symbol;Acc:HGNC:22997]
ENSG00000117122	MFAP2	microfibril associated protein 2 [Source:HGNC Symbol;Acc:HGNC:7033]
ENSG00000268941	LINC01711	long intergenic non-protein coding RNA 1711 [Source:HGNC Symbol;Acc:HGNC:28663]
ENSG00000139278	GLIPR1	GLI pathogenesis related 1 [Source:HGNC Symbol;Acc:HGNC:17001]
ENSG00000166033	HTRA1	HtrA serine peptidase 1 [Source:HGNC Symbol;Acc:HGNC:9476]
ENSG00000183098	GPC6	glypican 6 [Source:HGNC Symbol;Acc:HGNC:4454]
ENSG00000138080	EMILIN1	elastin microfibril interfacer 1 [Source:HGNC Symbol;Acc:HGNC:19880]
ENSG00000105664	COMP	cartilage oligomeric matrix protein [Source:HGNC Symbol;Acc:HGNC:2227]
ENSG00000111817	DSE	dermatan sulfate epimerase [Source:HGNC Symbol;Acc:HGNC:21144]
ENSG00000127083	OMD	osteomodulin [Source:HGNC Symbol;Acc:HGNC:8134]
ENSG00000279030		
ENSG00000118523	CCN2	cellular communication network factor 2 [Source:HGNC Symbol;Acc:HGNC:2500]
ENSG00000150093	ITGB1	integrin subunit beta 1 [Source:HGNC Symbol;Acc:HGNC:6153]
ENSG00000100934	SEC23A	Sec23 homolog A, coat complex II component [Source:HGNC Symbol;Acc:HGNC:10701]
ENSG00000226012	AP001434.1	novel transcript
ENSG00000135074	ADAM19	ADAM metallopeptidase domain 19 [Source:HGNC Symbol;Acc:HGNC:197]
ENSG00000248458	AL139147.1	novel transcript, antisense to SGIP1
ENSG00000161638	ITGA5	integrin subunit alpha 5 [Source:HGNC Symbol;Acc:HGNC:6141]
ENSG00000100097	LGALS1	galectin 1 [Source:HGNC Symbol;Acc:HGNC:6561]
ENSG00000234703	AF015262.1	novel transcript
ENSG00000235649	MXRA5Y	matrix remodeling associated 5 Y-linked (pseudogene) [Source:HGNC Symbol;Acc:HGNC:23932]
ENSG00000107796	ACTA2	actin, alpha 2, smooth muscle, aorta [Source:HGNC Symbol;Acc:HGNC:130]
ENSG00000174099	MSRB3	methionine sulfoxide reductase B3 [Source:HGNC Symbol;Acc:HGNC:27375]
ENSG00000091986	CCDC80	coiled-coil domain containing 80 [Source:HGNC Symbol;Acc:HGNC:30649]
ENSG00000162849	KIF26B	kinesin family member 26B [Source:HGNC Symbol;Acc:HGNC:25484]
ENSG00000103196	CRISPLD2	cysteine rich secretory protein LCCL domain containing 2 [Source:HGNC Symbol;Acc:HGNC:25248]
ENSG00000176971	FIBIN	fin bud initiation factor homolog [Source:HGNC Symbol;Acc:HGNC:33747]
ENSG00000266995	AP001542.1	telomeric repeat binding factor (NIMA-interacting) 1 (TERF1) pseudogene
ENSG00000249406	AC015909.1	novel transcript
ENSG00000155254	MARVELD1	MARVEL domain containing 1 [Source:HGNC Symbol;Acc:HGNC:28674]
ENSG00000129038	LOXL1	lysyl oxidase like 1 [Source:HGNC Symbol;Acc:HGNC:6665]
ENSG00000084636	COL16A1	collagen type XVI alpha 1 chain [Source:HGNC Symbol;Acc:HGNC:2193]
ENSG00000182667	NTM	neurotrimin [Source:HGNC Symbol;Acc:HGNC:17941]
ENSG00000145423	SFRP2	secreted frizzled related protein 2 [Source:HGNC Symbol;Acc:HGNC:10777]
ENSG00000136859	ANGPTL2	angiopoietin like 2 [Source:HGNC Symbol;Acc:HGNC:490]

ENSG00000121904	CSMD2	CUB and Sushi multiple domains 2 [Source:HGNC Symbol;Acc:HGNC:19290]
ENSG00000151617	EDNRA	endothelin receptor type A [Source:HGNC Symbol;Acc:HGNC:3179]
ENSG00000180447	GAS1	growth arrest specific 1 [Source:HGNC Symbol;Acc:HGNC:4165]
ENSG00000133816	MICAL2	microtubule associated monooxygenase, calponin and LIM domain containing 2 [Source:HGNC Symbol;Acc:HGNC:24693]
ENSG00000189320	FAM180A	family with sequence similarity 180 member A [Source:HGNC Symbol;Acc:HGNC:33773]
ENSG00000166923	GREM1	gremlin 1, DAN family BMP antagonist [Source:HGNC Symbol;Acc:HGNC:2001]
ENSG00000171502	COL24A1	collagen type XXIV alpha 1 chain [Source:HGNC Symbol;Acc:HGNC:20821]
ENSG00000137801	THBS1	thrombospondin 1 [Source:HGNC Symbol;Acc:HGNC:11785]
ENSG00000196923	PDLIM7	PDZ and LIM domain 7 [Source:HGNC Symbol;Acc:HGNC:22958]
ENSG00000091136	LAMB1	laminin subunit beta 1 [Source:HGNC Symbol;Acc:HGNC:6486]
ENSG00000196611	MMP1	matrix metallopeptidase 1 [Source:HGNC Symbol;Acc:HGNC:7155]
ENSG00000229056	HECW2-AS1	HECW2 antisense RNA 1 [Source:HGNC Symbol;Acc:HGNC:54062]
ENSG00000122870	BICC1	BicC family RNA binding protein 1 [Source:HGNC Symbol;Acc:HGNC:19351]
ENSG00000162576	MXRA8	matrix remodeling associated 8 [Source:HGNC Symbol;Acc:HGNC:7542]
ENSG00000227359	AC017074.1	novel transcript
ENSG00000169756	LIMS1	LIM zinc finger domain containing 1 [Source:HGNC Symbol;Acc:HGNC:6616]
ENSG00000154553	PDLIM3	PDZ and LIM domain 3 [Source:HGNC Symbol;Acc:HGNC:20767]
ENSG00000167601	AXL	AXL receptor tyrosine kinase [Source:HGNC Symbol;Acc:HGNC:905]
ENSG00000089472	HEPH	hephaestin [Source:HGNC Symbol;Acc:HGNC:4866]
ENSG00000168386	FILIP1L	filamin A interacting protein 1 like [Source:HGNC Symbol;Acc:HGNC:24589]
ENSG00000174807	CD248	CD248 molecule [Source:HGNC Symbol;Acc:HGNC:18219]
ENSG00000048545	GUCA1A	guanylate cyclase activator 1A [Source:HGNC Symbol;Acc:HGNC:4678]
ENSG00000101335	MYL9	myosin light chain 9 [Source:HGNC Symbol;Acc:HGNC:15754]
ENSG00000121297	TSHZ3	teashirt zinc finger homeobox 3 [Source:HGNC Symbol;Acc:HGNC:30700]
ENSG00000175509	AL078621.2	acrosin (ACR) pseudogene
ENSG00000116774	OLFML3	olfactomedin like 3 [Source:HGNC Symbol;Acc:HGNC:24956]
ENSG00000122691	TWIST1	twist family bHLH transcription factor 1 [Source:HGNC Symbol;Acc:HGNC:12428]
ENSG00000167460	TPM4	tropomyosin 4 [Source:HGNC Symbol;Acc:HGNC:12013]
ENSG00000107957	SH3PXD2A	SH3 and PX domains 2A [Source:HGNC Symbol;Acc:HGNC:23664]
ENSG00000130254	SAFB2	scaffold attachment factor B2 [Source:HGNC Symbol;Acc:HGNC:21605]
ENSG00000106080	FKBP14	FKBP prolyl isomerase 14 [Source:HGNC Symbol;Acc:HGNC:18625]
ENSG00000183671	GPR1	G protein-coupled receptor 1 [Source:HGNC Symbol;Acc:HGNC:4463]
ENSG00000182326	C1S	complement C1s [Source:HGNC Symbol;Acc:HGNC:1247]
ENSG00000105989	WNT2	Wnt family member 2 [Source:HGNC Symbol;Acc:HGNC:12780]
ENSG00000130508	PXDN	peroxidasin [Source:HGNC Symbol;Acc:HGNC:14966]
ENSG00000198768	APCDD1L	APC down-regulated 1 like [Source:HGNC Symbol;Acc:HGNC:26892]
ENSG00000128656	CHN1	chimerin 1 [Source:HGNC Symbol;Acc:HGNC:1943]
ENSG00000260517	AC009093.2	novel transcript
ENSG00000151790	TDO2	tryptophan 2,3-dioxygenase [Source:HGNC Symbol;Acc:HGNC:11708]
ENSG00000140931	CMTM3	CKLF like MARVEL transmembrane domain containing 3 [Source:HGNC Symbol;Acc:HGNC:19174]
ENSG00000251085		
ENSG00000177234	LINC01561	long intergenic non-protein coding RNA 1561 [Source:HGNC Symbol;Acc:HGNC:31365]

Table S5. CXCR4- (A) and FAP-specific (B) gene signatures analyzed using the "investigate gene sets" module of the Gene Set Enrichment Analysis (GSEA) webpage. FDR: false discovery rate.

A CXCR4

Gene Set Name	# Genes in Overlap	FDR / *q* value
HALLMARK_INFLAMMATORY_RESPONSE [200]	16	1.46 e^{-14}
HALLMARK_ALLOGRAFT_REJECTION [200]	15	1.67 e^{-13}
HALLMARK_IL2_STAT5_SIGNALING [199]	9	2.06 e^{-6}
HALLMARK_KRAS_SIGNALING_UP [200]	9	2.06 e^{-6}
HALLMARK_IL6_JAK_STAT3_SIGNALING [87]	6	1.23 e^{-5}
HALLMARK_COMPLEMENT [200]	8	1.23 e^{-5}
HALLMARK INTERFERON GAMMA_RESPONSE [200]	8	1.23 e^{-5}
HALLMARK_TNFA_SIGNALING_VIA_NFKB [200]	8	1.23 e^{-5}
HALLMARK_HYPOXIA [200]	5	8.25 e^{-3}
HALLMARK_PI3K_AKT_MTOR_SIGNALING [105]	3	4.73 e^{-2}

B FAP

Gene Set Name	# Genes in Overlap	FDR / *q* value
HALLMARK_EPITHELIAL_MESENCHYMAL_ TRANSITION [200]	62	2.18 e^{-98}
HALLMARK_COAGULATION [138]	15	1.37 e^{-15}
HALLMARK_APICAL_JUNCTION [200]	11	2.39 e^{-8}
HALLMARK_ANGIOGENESIS [36]	6	1.34 e^{-7}
HALLMARK_MYOGENESIS [200]	10	2.02 e^{-7}
HALLMARK_INFLAMMATORY_RESPONSE [200]	8	2.43 e^{-5}
HALLMARK_UV_RESPONSE_DN [144]	7	2.45 e^{-5}
HALLMARK_HYPOXIA [200]	7	1.82 e^{-4}
HALLMARK_APOPTOSIS [161]	6	4.3 e^{-4}
HALLMARK_COMPLEMENT [200]	6	1.15 e^{-3}

Figure S1. Spearman correlation coefficients of CXCR4 expression and infiltration of B cells (**a**), monocytes (**b**) and macrophages (**c**) within TCGA database. Analyses were performed with TIMER2.0 web resource. Color-filled boxes represent significant results ($p \leq 0.05$) – with red fillings indicating positive and blue fillings indicating negative correlation coefficients.

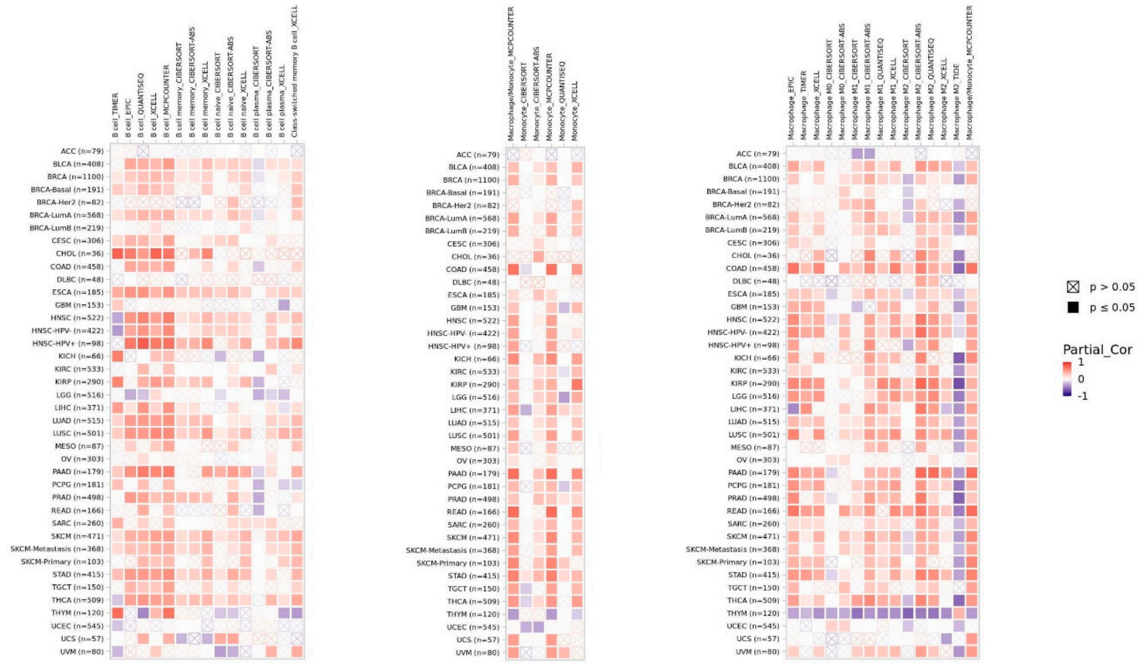

(**a**) (**b**) (**c**)

Figure S2. Pearson R values between FAP expression and angiogenesis-related genes within the TCGA pan-cancer cohort.

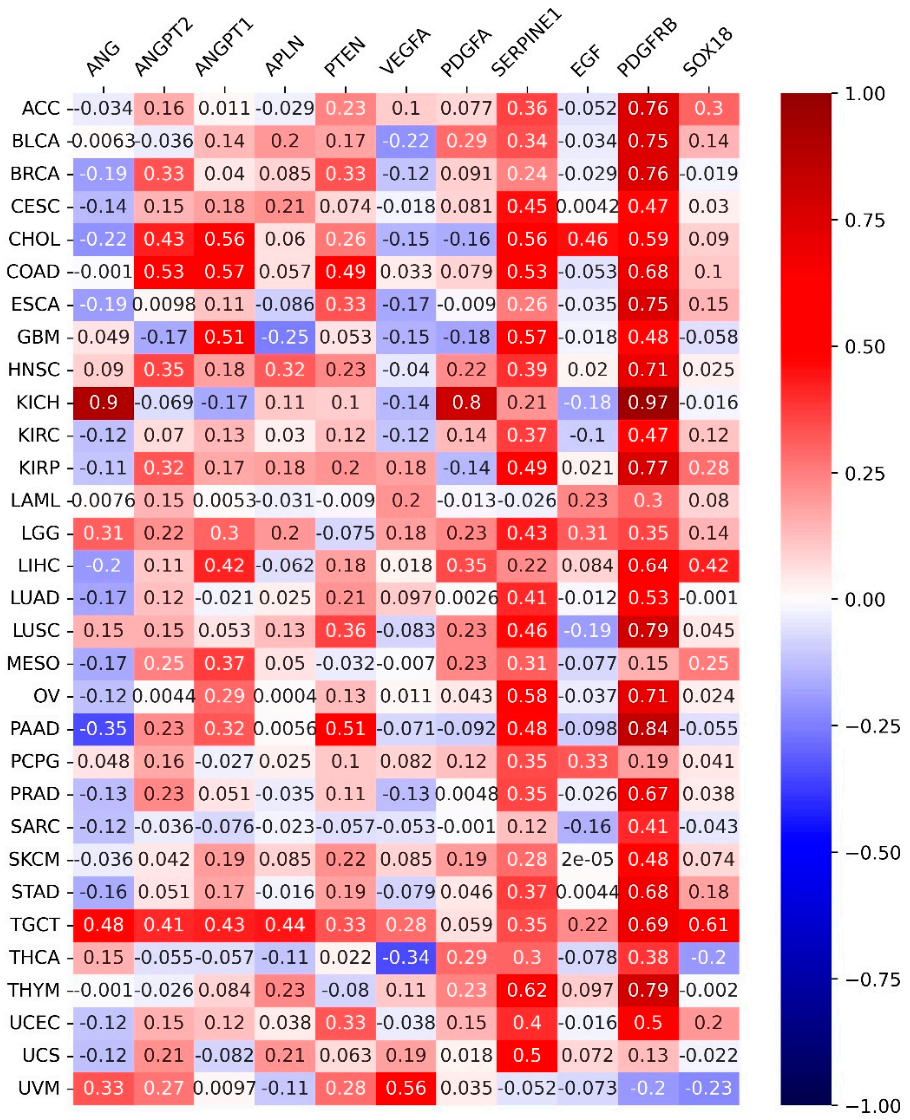

Figure S3. Protein expression depending on CXCR4/FAP high vs. low expression in the TCGA database. PD-L1 (**a**) and CTLA4 (**b**) were measured according to CXCR4 expression. VEGFR2 (**c**), HIF1A (**d**) and ETS1 (**e**) were measured according to FAP expression.

CXCR4

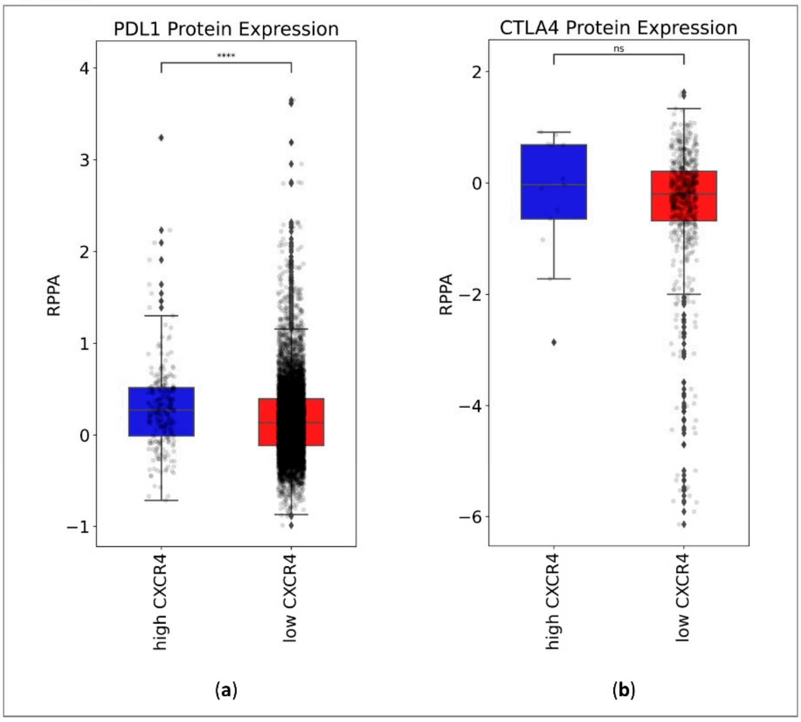

FAP

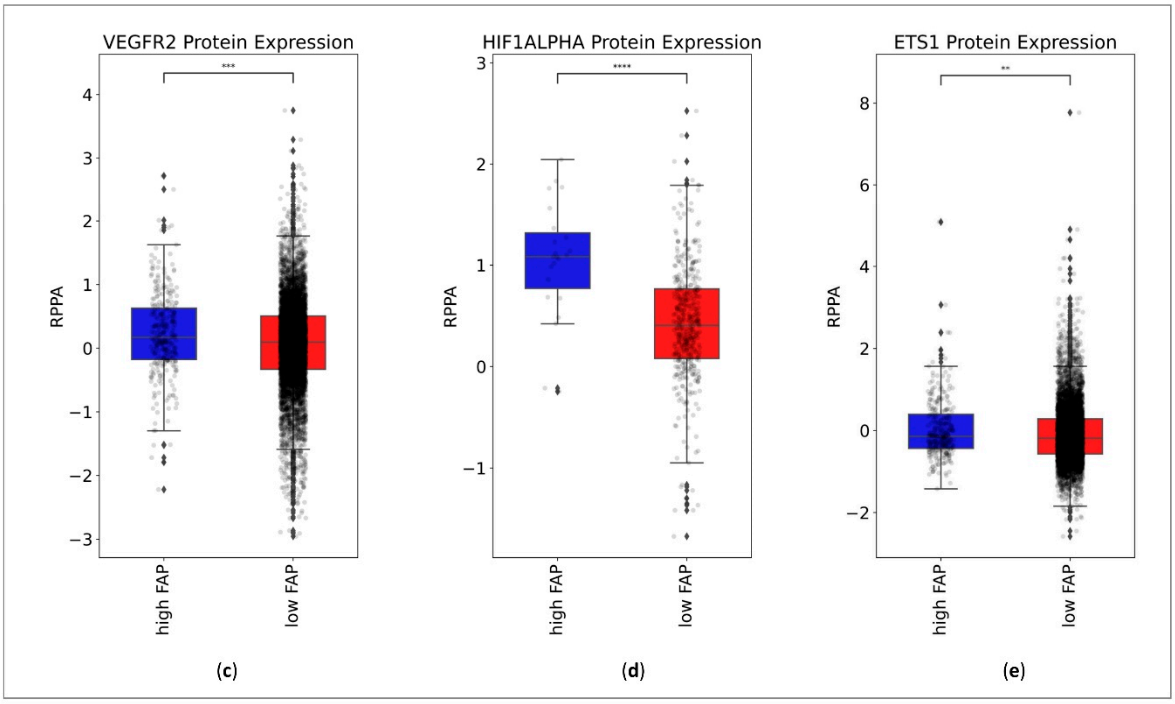

Figure S4. Cell-type specific expression of CXCR4 and a selection of immune-related genes within a single cell sequencing dataset representing head and neck cancer (GSE103322).

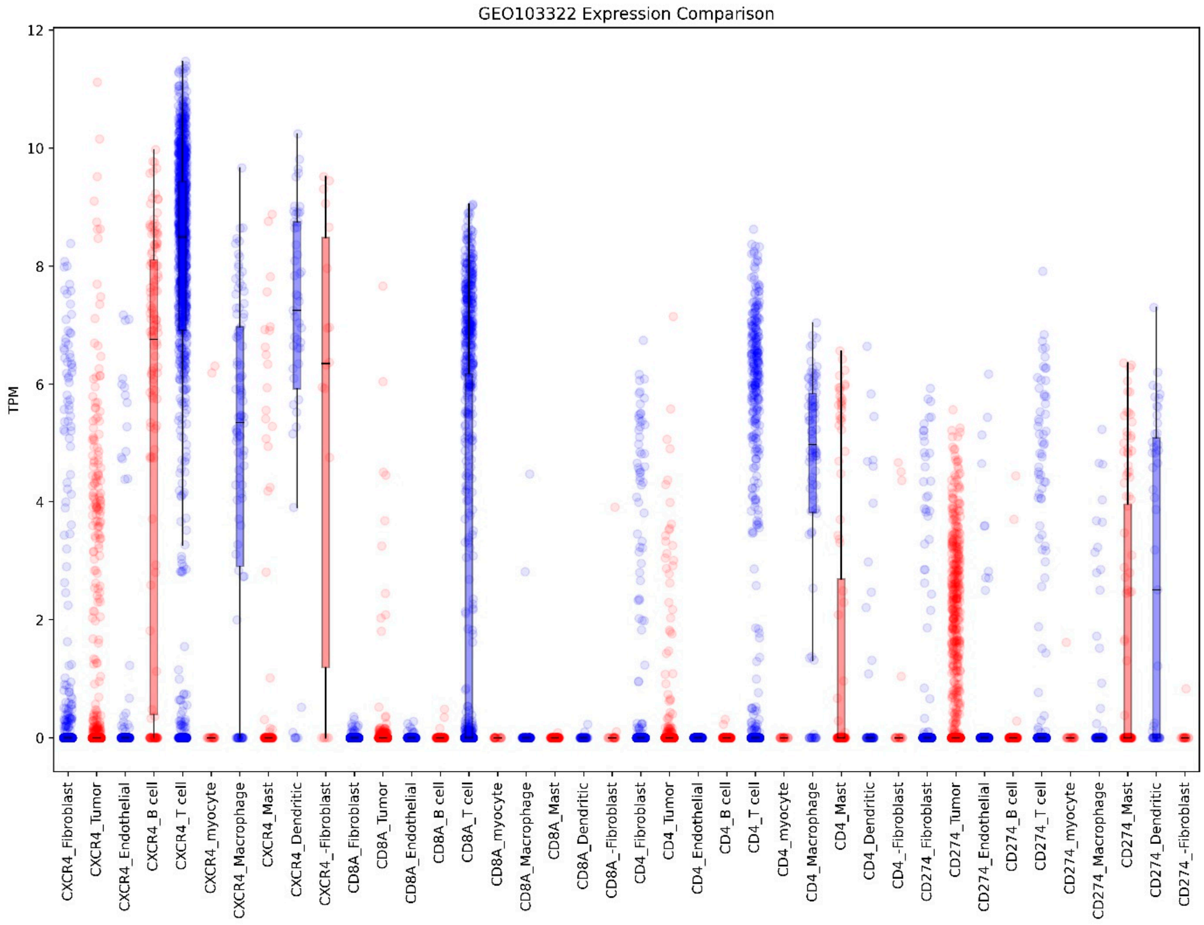

Figure S4. Cell-type specific expression of FAP and a selection of angiogenesis-related genes within a single cell sequencing dataset representing head and neck cancer (GSE103322).

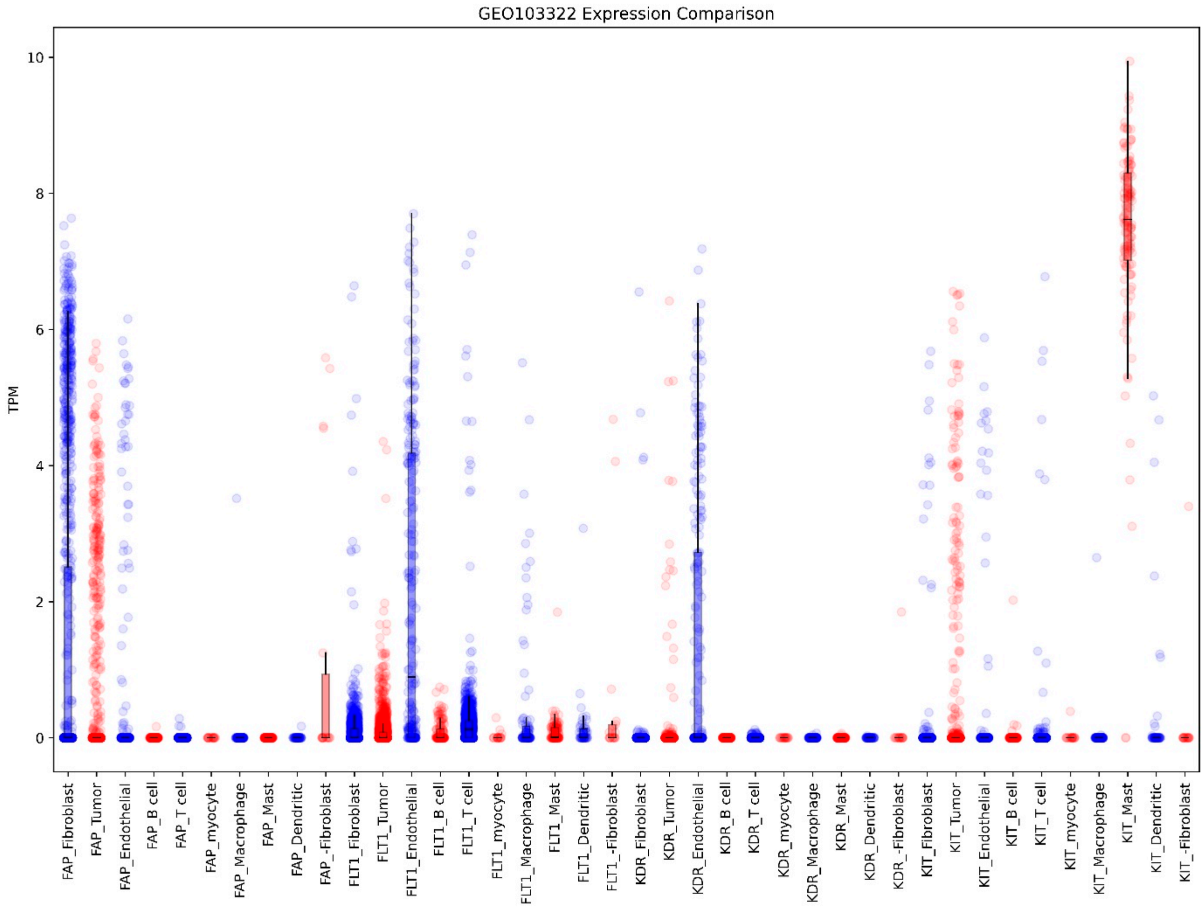

Supplement: Supplementary file 1 [file cancers-15-00392-s001.zip › cancers-2003118-Supplementary.pdf]
