# Supplementary figures and images for: Predicting Microenvironment in CXCR4- and FAP-Positive Solid Tumors—A Pan-Cancer Machine Learning Workflow for Theranostic Target Structures
Source: Cancers (Basel). 2023 Jan 6;15(2):392. doi: 10.3390/cancers15020392 (PMC9856808; doi:10.3390/cancers15020392)

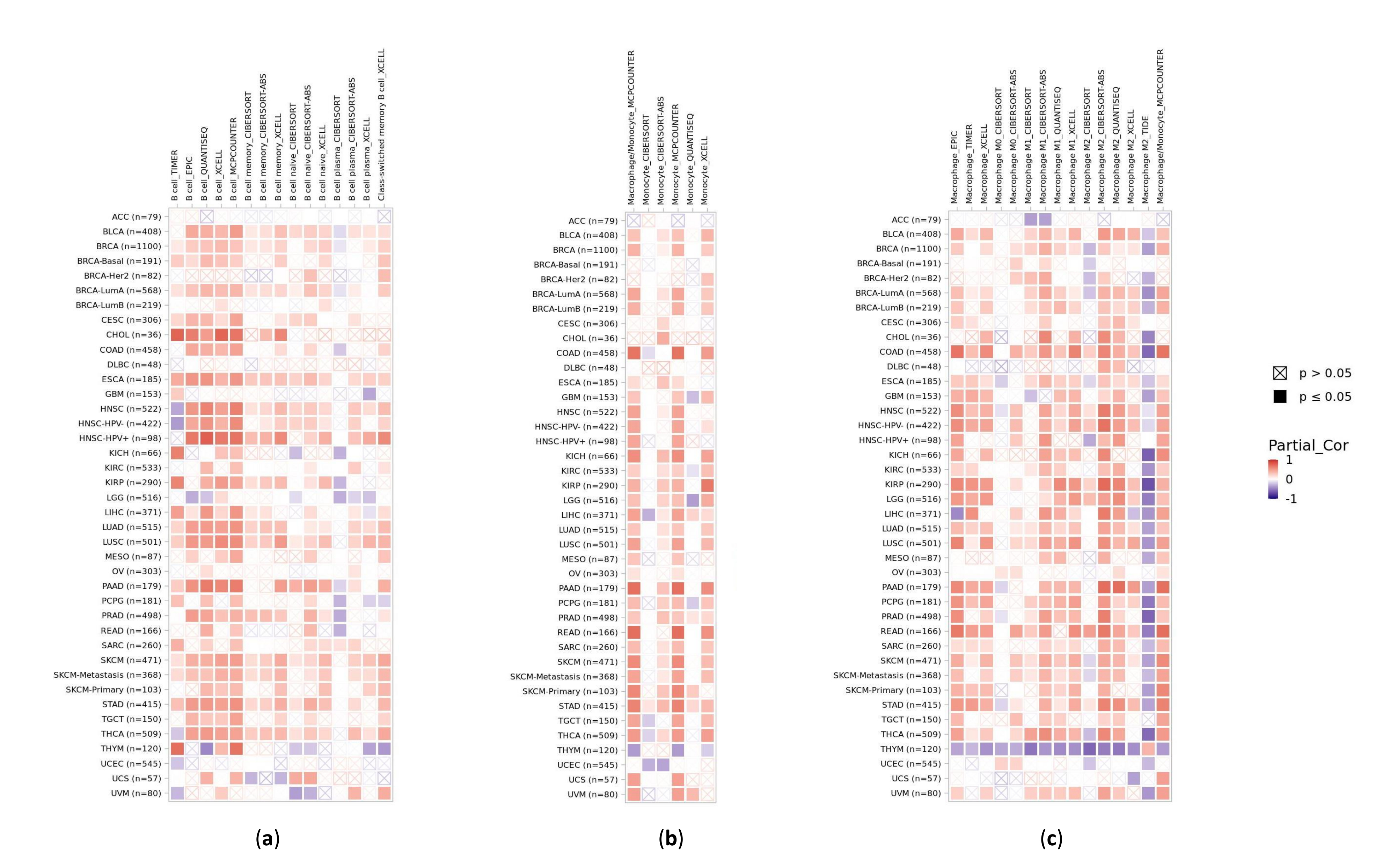

Supplement: Supplementary file 1 [file cancers-15-00392-s001.zip › FigureS1_Cancers.jpg]

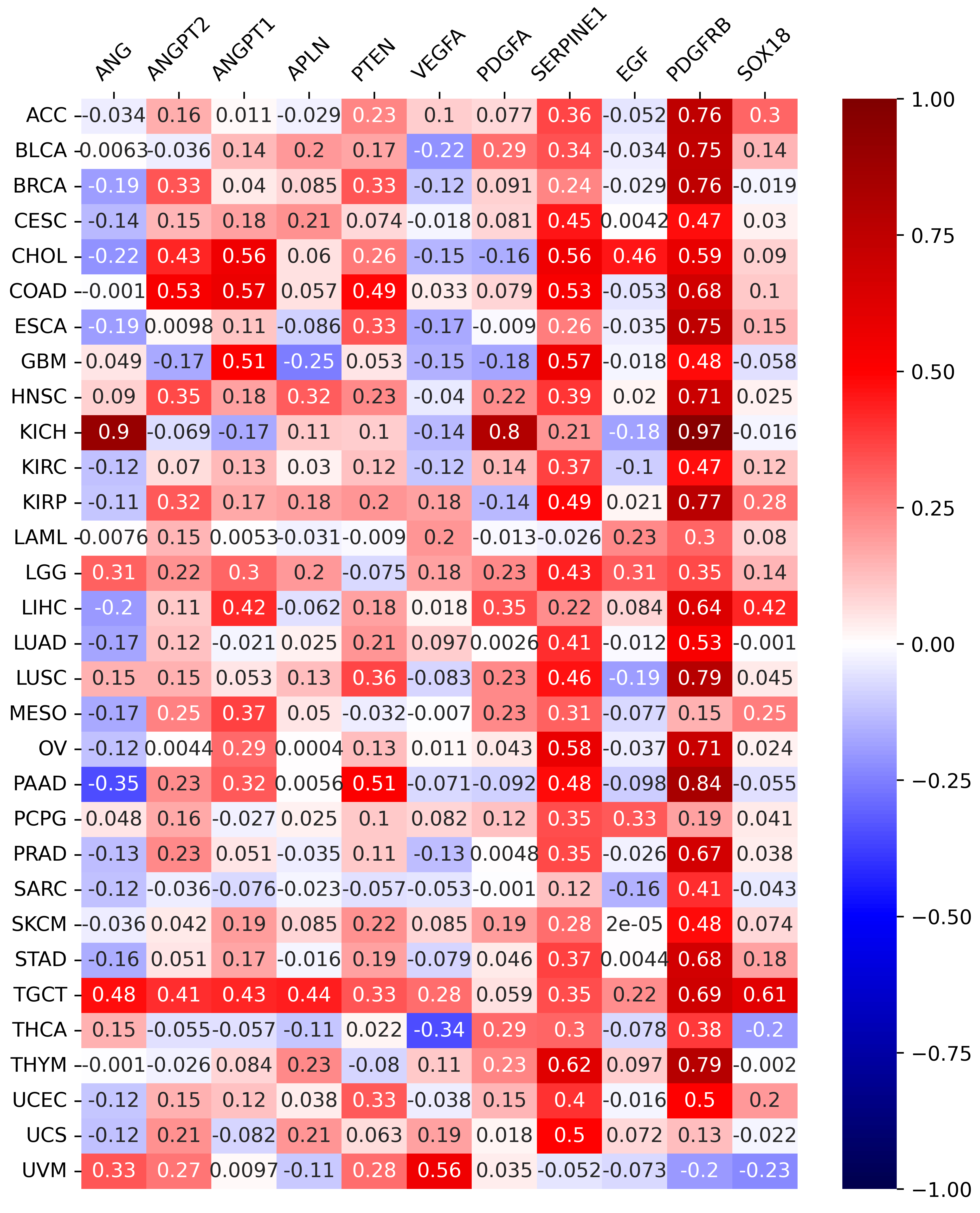

Supplement: Supplementary file 1 [file cancers-15-00392-s001.zip › FigureS2_Cancers.jpg]

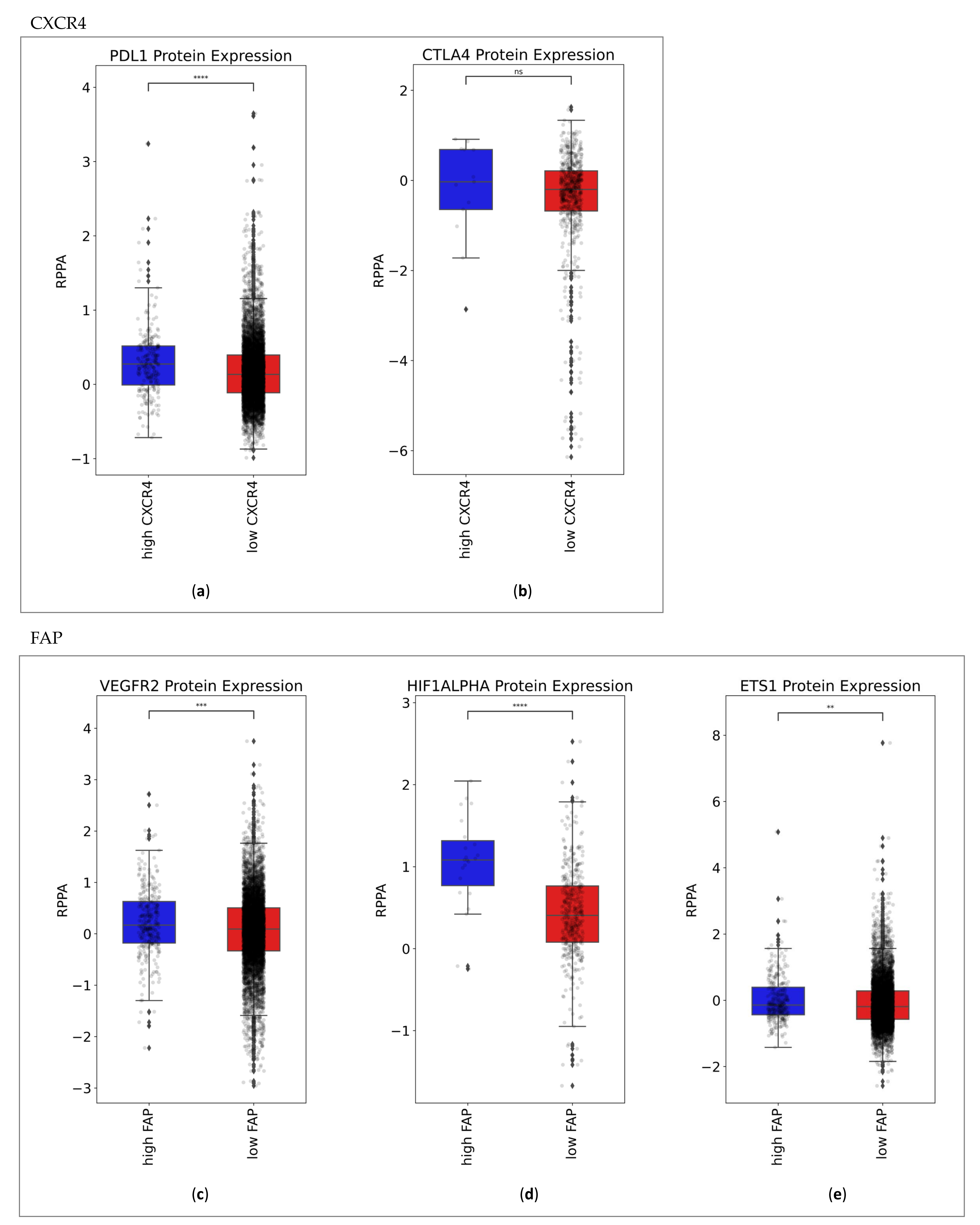

Supplement: Supplementary file 1 [file cancers-15-00392-s001.zip › FigureS3_Cancers.jpg]

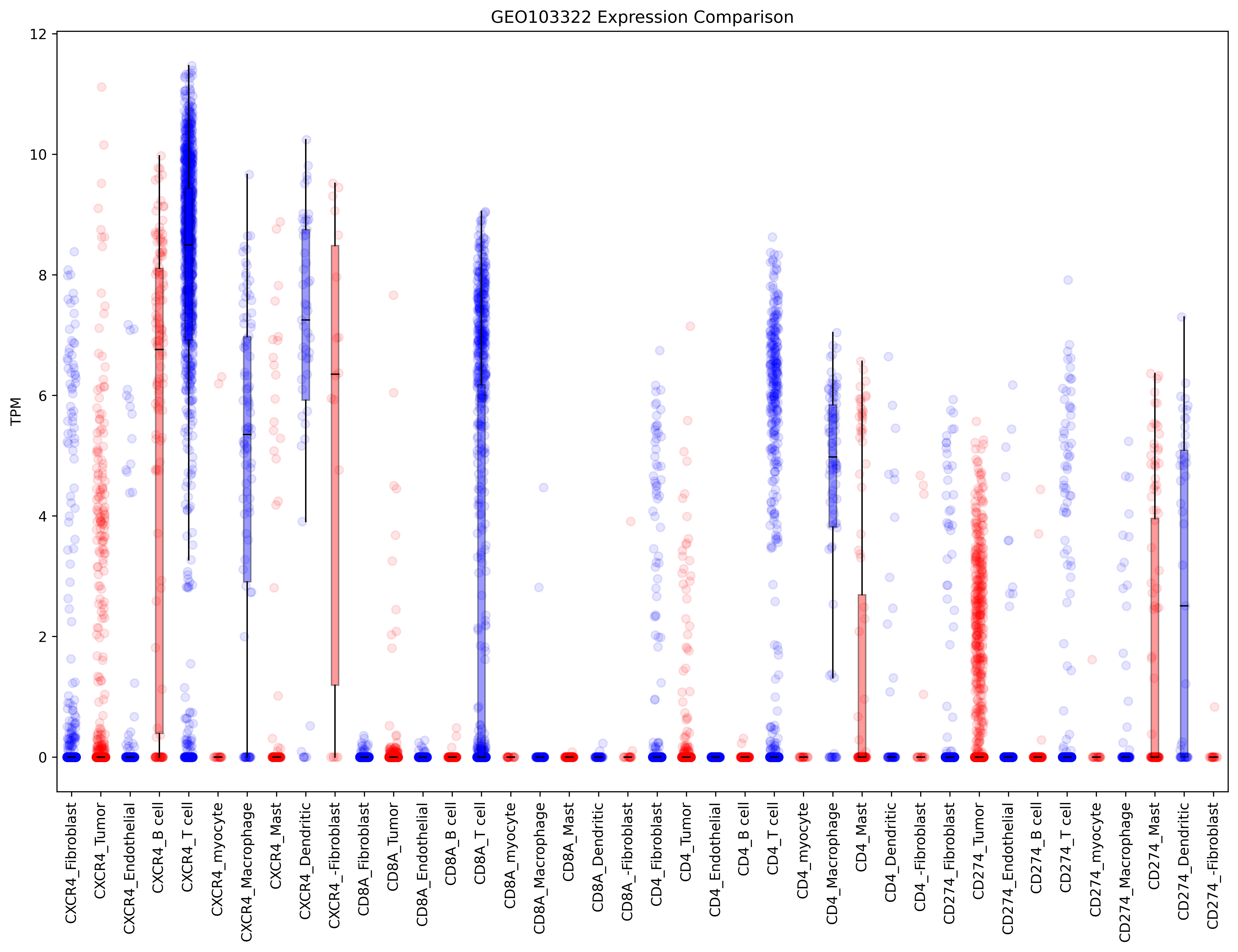

Supplement: Supplementary file 1 [file cancers-15-00392-s001.zip › FigureS4_Cancers.jpg]

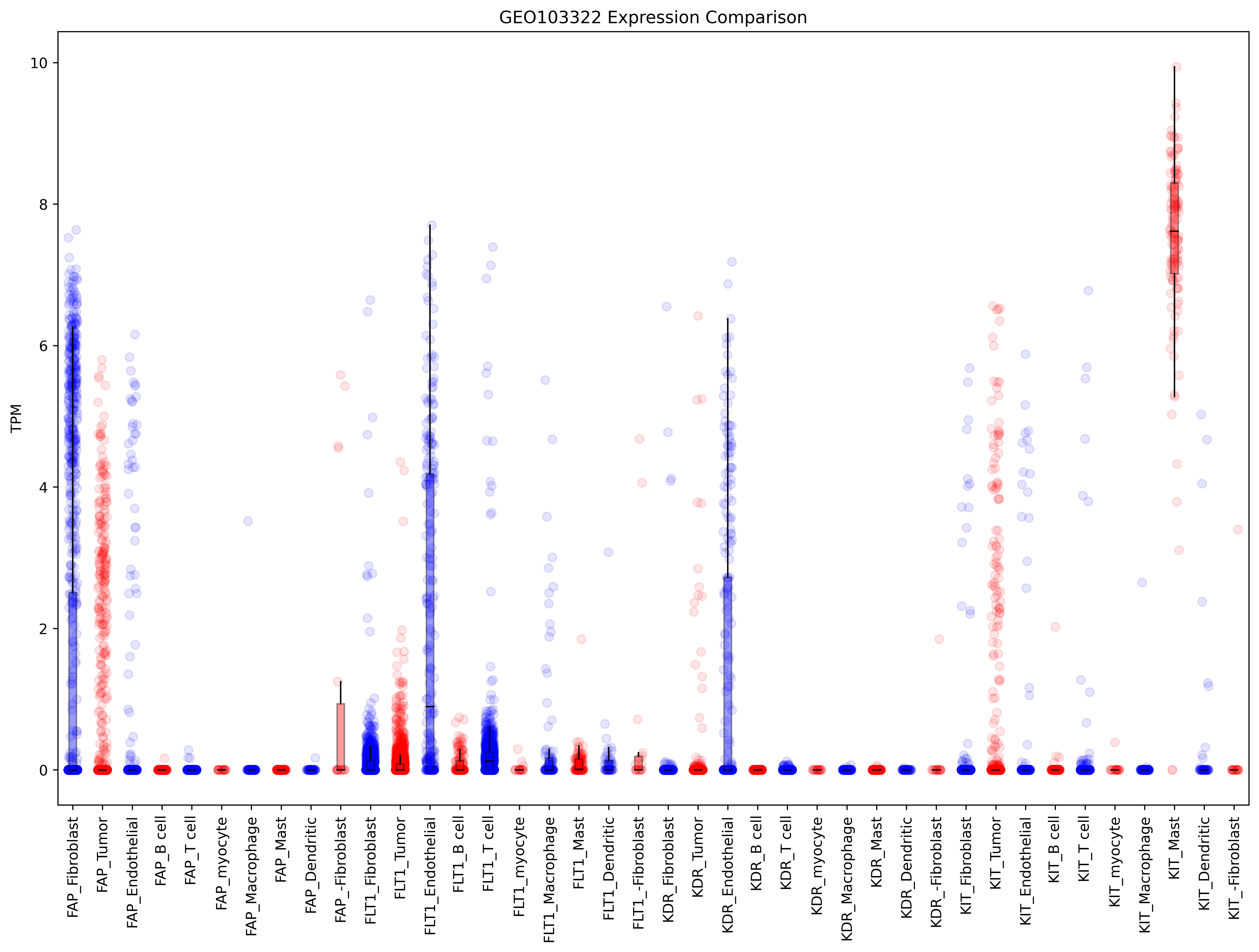

Supplement: Supplementary file 1 [file cancers-15-00392-s001.zip › FigureS5_Cancers.jpg]
